# Assessing Biosynthetic Gene Cluster Diversity of Specialized Metabolites in the Conserved Gut Symbionts of Herbivorous Turtle Ants

**DOI:** 10.3389/fmicb.2021.678100

**Published:** 2021-06-29

**Authors:** Anaïs Chanson, Corrie S. Moreau, Christophe Duplais

**Affiliations:** ^1^Université de Guyane, UMR 8172 EcoFoG, AgroParisTech, CNRS, Cirad, INRAE, Université des Antilles, Kourou, France; ^2^Department of Entomology and Ecology & Evolutionary Biology, Cornell University, Ithaca, NY, United States; ^3^CNRS, UMR 8172 EcoFoG, AgroParisTech, Cirad, INRAE, Université des Antilles, Université de Guyane, Kourou, France

**Keywords:** insect-microbe mutualism, ants, metagemonic, biosynthetic gene cluster, gut bacteria, *Cephalotes*

## Abstract

*Cephalotes* are herbivorous ants (>115 species) feeding on low-nitrogen food sources, and they rely on gut symbionts to supplement their diet by recycling nitrogen food waste into amino acids. These conserved gut symbionts, which encompass five bacterial orders, have been studied previously for their primary nitrogen metabolism; however, little is known about their ability to biosynthesize specialized metabolites which can play a role in bacterial interactions between communities living in close proximity in the gut. To evaluate the biosynthetic potential of their gut symbionts, we mine 14 cultured isolate genomes and gut metagenomes across 17 *Cephalotes* species to explore the biodiversity of biosynthetic gene clusters (BGCs) producing specialized metabolites. The diversity of BGCs across *Cephalotes* phylogeny was analyzed using sequence similarity networking and BGC phylogenetic reconstruction. Our results reveal that the conserved gut symbionts involved in the nutritional symbiosis possess 80% of all the 233 BGCs retrieved in this work. Furthermore, the phylogenetic analysis of BGCs reveals different patterns of distribution, suggesting different mechanisms of conservation. A siderophore BGC shows high similarity in a single symbiont across different ant host species, whereas a BGC encoding the production of non-ribosomal peptides (NRPs) found different symbionts within a single host species. Additionally, BGCs were abundant in four of the five bacterial orders of conserved symbionts co-occurring in the hindgut. However, one major symbiont localized alone in the midgut lack BGCs. Because the spatial isolation prevents direct interaction with other symbionts, this result supports the idea that BGCs are maintained in bacteria living in close proximity but are dispensable for an alone-living symbiont. These findings together pave the way for studying the mechanisms of BGC conservation and evolution in gut bacterial genomes associated with *Cephalotes*. This work also provides a genetic background for further study, aiming to characterize bacterial specialized metabolites and to understand their functional role in multipartite mutualisms between conserved gut symbionts and *Cephalotes* turtle ants.

## Introduction

Bacterial symbionts are widespread among insects and have shaped the evolution of their hosts (Hosokawa et al., 2006; Jiggins and Hurst, 2011). The symbiotic interactions between bacterial communities inside the host, as well as between bacteria and hosts, rely on primary and secondary metabolites, metabolic pathways, and the enzymes that regulate metabolic flux.

Among mutualistic bacteria, the nutritional symbionts supply a large diversity of nutrients to the diet of their insect hosts, which often feed on nutritionally limited food sources. In the case of strictly bloodsucking ticks, intracellular bacterial symbionts provide vitamin B to the host (Duron et al., 2018; Binetruy et al., 2020). Herbivorous aphids and ants feeding on nitrogen-poor diets depend on gut bacteria to recycle nitrogen-rich food waste to biosynthesize essential and non-essential amino acids (Hansen and Moran, 2011; Hu et al., 2018). Gut bacteria are also essential to degrade various substrates to enrich the host’s diet as is the case for xylophagous termites that rely on gut bacteria to degrade the lignocellulose (Boucias et al., 2013), whereas the gut bacterial community of bees degrades polysaccharides (Zheng et al., 2019).

Another type of microbial symbiosis involves defensive mechanisms as the bacterial symbionts can enhance insect resistance to a variety of natural enemies including bacteria, fungi, or nematodes (Scarborough et al., 2005; Hedges et al., 2008). For example, in different insect families, including beetles, wasps, bees, and ants, the bacterial symbionts produce a cocktail of antimicrobial compounds to protect the nest (Chevrette et al., 2019), the brood (Engl et al., 2018; Flórez et al., 2018), or a fungal strain in case of multipartite mutualisms (Barke et al., 2010; Matarrita-Carranza et al., 2017).

Bacterial specialized metabolites are produced *via* specific groups of genes called biosynthetic gene clusters (BGCs). In a BGC, genes are located in close proximity to each other in the bacterial genome and encode for specific enzymatic steps in the biosynthetic pathway of metabolites (Ziemert et al., 2016). Bioinformatics tools have been developed to mine bacterial genomes and to retrieve the BGCs of different chemical families including aryl polyene, lanthipeptide, non-ribosomal peptide (NRP), polyketide (PK), and terpene (Ziemert et al., 2016; Adamek et al., 2017). Metabolites play several roles in bacterial communities for colonizing tissue, communication through quorum sensing, competition using antimicrobial molecules, and nutrient acquisition with siderophore (Zhang et al., 2009; Chaturvedi et al., 2012; Shevchuk et al., 2014; Schöner et al., 2016). BGC studies often focus on the genomes of culturable strains from environmental bacteria (Paulus et al., 2017; Crits-Christoph et al., 2018) or host-associated bacteria (Kroiss et al., 2010; Beemelmanns et al., 2016) and contribute to the discovery of bioactive compounds for medicine. Mining of bacterial metagenomes has also been investigated from environmental samples (Cuadrat et al., 2018), as well as bacterial communities associated within hosts (Donia et al., 2011; Wilson and Piel, 2013; Altamia et al., 2020; Waterworth et al., 2020).

The study of bacterial BGCs in symbiosis aims to predict the chemical structure of specialized metabolites encoded in symbiont genomes and provide a framework to identify candidate genes which are conserved to support bacterial colonization and maintenance within a host. In addition, the comparison between bacterial BGCs across different hosts (Ashen and Goff, 2000) in different geographic areas (Edlund et al., 2011) allows us to test what environmental, ecological, or phylogenetic factors shape BGC diversity in host-associated bacterial communities.

To investigate the BGC diversity of insect symbionts, we studied the association between gut bacteria and herbivorous *Cephalotes* turtle ants. In this nutritional symbiosis, the nitrogen-poor diet of ants is supplemented by a core microbiome which recycles urea food waste into amino acids beneficial to the ant (Russell et al., 2009; Duplais et al., 2021). Genomic approaches have revealed the redundancy in functions related to the nitrogen flux in the genome of conserved symbionts belonging to five bacterial orders (Hu et al., 2018). Unlike most intracellular or extracellular single nutritional symbionts, the *Cephalotes* multipartite gut bacterial mutualisms may retain metabolic functions selected not solely for direct benefit to hosts but also for maintaining diverse bacterial community members. To assess if the gut bacteria associated with *Cephalotes* possess BGCs, we focused on 14 bacterial genomes and metagenomes from the gut of 17 *Cephalotes* species (Hu et al., 2018). Here, we mined both genomes and metagenomes to BGCs encoding the specialized metabolites. We analyze the bacterial origin of each BGC to understand their diversity and occurrence across gut symbionts and ant host species. Using sequence similarity networking and BGC phylogenetic reconstruction, we focused on the most abundant BGCs (aryl polyene, NRP, PK, and siderophore) to identify architectural similarity patterns across different symbionts and host species. This work highlights the relevance of genome and metagenome mining in insect–microbe symbiosis and paves the way for future studies on the evolution of BGCs in symbiont genomes across the host phylogeny and the role of specialized metabolites in the multipartite mutualisms between gut symbionts and turtle ants.

## Materials and Methods

### Genome and Metagenome Analyses

The 14 genomes of cultured gut bacteria and 18 metagenomes of *Cephalotes* gut bacteria were obtained from JGI-IMG version 5.0 (Chen et al., 2019; Supplementary Tables 1, 2) from the previous projects Gs0085494 (“*Cephalotes varians* microbial communities from the Florida Keys, United States”), Gs0114286 (“Symbiotic bacteria isolated from *Cephalotes varians*”), Gs0117930 (“*Cephalotes* ants gut microbiomes”), and Gs0118097 (“Symbiotic bacteria isolated from *Cephalotes rohweri*”) (Hu et al., 2018).

The 14 cultured isolate genomes are all part of the *Cephalotes* core microbiome: two *Cephaloticoccus* genomes (order: Opitutales, class: Opitutae; one genome from each ant host), four *Ventosimonas* genomes (order: Pseudomonadales, class: Gammaproteobacteria; one genome from *C. varians* and three genomes from *C. rohweri*), six Burkholderiales genomes (class: Betaproteobacteria; four genomes from *C. varians* and two genomes from *C. rohweri*), one Rhizobiales genome (class: Alphaproteobacteria; from *C. varians*), and one Xanthomonadales genome (class: Gammaproteobacteria; from *C. rohweri*). All these bacteria belong to the Proteobacteria phylum, except Opitutales, which is part of the Verrucomicrobia phylum.

The metagenomes were analyzed *via* the software Anvi’o version 5.5 (Eren et al., 2015) to sort the different bacterial families composing each metagenome into distinct bins. In this analysis, the sequences of a metagenome in fasta format are used to create a contig database and a profile database. Then, this contig database is visualized, and bins are manually created to maximize completeness while minimizing contamination. Finally, the software CheckM version 1.1 (Parks et al., 2015) was used to verify the completeness, contamination, and strain homogeneity of each bin and to identify the taxonomic lineage of each bin (Supplementary Figure 1 and Supplementary Table 3).

The published *Cephalotes* phylogeny was retrieved (Price et al., 2014), and the packages ape (Paradis et al., 2004) and phytools (Revell, 2012) of R software version 3.6.1 (R Core Team, 2019) were used to exclude from this phylogeny the *Cephalotes* species from which no symbiont genomes or metagenomes were available.

### Bacterial BGC Analysis

The bacterial BGCs of each genome and each metagenomic bin (group of contigs assigned to individual genome) were analyzed with antiSMASH version 5.0 (Blin et al., 2019; Supplementary Figure 1 and Supplementary Tables 4, 5) with the following analysis options: strict detection and activation of search for KnownClusterBlast, ClusterBlast, SubClusterBlast, and Active site finder. BGCs smaller than 5 kb were then filtered out of the data and were not included. The taxonomic classification of each cluster was verified to the genus level using the software Blast+ (Altschul et al., 1990; Supplementary Table 5). There was no identification conflict between CheckM and Blast+. NaPDos (https://npdomainseeker.sdsc.edu/, accessed July 2020) (Ziemert et al., 2012) was used to classify the ketosynthase (KS) domain and condensation (C) domain sequences of NRP and PK retrieved from the genome and metagenome mining analyses and to infer the KS and C phylogenies.

### Genomic Similarity Assessment

The genomic similarity between the cultured isolate genomes and the metagenomes from the *C. rohweri* and *C. varians* was assessed using two independent methods (Supplementary Tables 6, 7). First, each metagenomic bin was compared to each cultured isolate genome from the corresponding host species using the combined values of the average nucleotide identity (gANI) and alignment fraction (AF) (Konstantinidis and Tiedje, 2005; Varghese et al., 2015). In this method, the nucleotide sequences of the protein-coding genes of each metagenomic bin (genome 1) and each cultured isolate genome (genome 2) are compared using NSimScan (Novichkov et al., 2016), and only the bidirectional best hits (BBHs) displaying at least 70% sequence identity over at least 70% of the length of the shorter sequence are kept. The gANI is calculated by dividing the sum of the percentage of identity times the alignment length for all BBHs by the sum of the lengths of the BBH genes. A gANI higher than 0.95 indicates a threshold for the same species level (Wang et al., 2016). The AF is calculated by dividing the sum of the lengths of all BBH genes by the sum of the length of all the genes in genome 1. For AF, values higher than 0.5 are considered conspecifics (Altamia et al., 2020).

Next, the contigs of each metagenomic bin were mapped to each cultured isolate genome from the corresponding host species using the bbmap.sh function in the BBMerge software version 38.22 (Bushnell et al., 2017) with the following parameters: kfilter = 22, subfilter = 15, and maxindel = 80. These parameters were used in a similar study published recently (Altamia et al., 2020). BBMap aligns contig sequences from each metagenomic bin (genome 1) and each cultured isolate genome (genome 2) and returns the percentage of contigs in genome 2 which were found in genome 1.

### Sequence Similarity Networking

The sequence similarity networking of the genomic and metagenomic bacterial BGCs was generated using the “biosynthetic gene similarity clustering and prospecting engine” BiG-SCAPE version 1.0.0 (Navarro-Munoz et al., 2020) (Supplementary Figure 1). The DNA sequences from each BGC were extracted from the AntiSMASH.gbk file output, and the similarity analysis was run using those extracted sequences. The network was constructed with the three following options: “–include_singletons,” “—mix,” and “–cutoffs 1.0.” Briefly, the Pfam domains of each BGC were identified using HMMER version 3.1b2 (Finn et al., 2011) with the respective hidden Markov models (HMMs) obtained from the Pfam database (Bateman et al., 2004). The distance metric is created by calculating the distance between every pair of BGC. Pairwise distance calculation is divided between three values: the percentage of shared domain types (Jaccard index), the similarity of aligned domain sequences (domain sequence similarity index), and the similarity of domain pair types (adjacency index). Specific details for each index and the method are available in Navarro-Munoz et al. (2020). The resulting similarity matrix was filtered with different thresholds between 0.4 and 0.2 (Cimermancic et al., 2014; Schorn et al., 2016; Adamek et al., 2018; Navarro-Munoz et al., 2020), and the threshold 0.45 was chosen because it filtered out enough to form different groups while maximizing the number of bacterial BGCs in each network (Cimermancic et al., 2014; Adamek et al., 2018). The filtered similarity matrix was visualized with Cytoscape version 3.7.2 (Shannon et al., 2003), using the MCL clusterization algorithm from clusterMaker2 (Morris et al., 2011) version 1.3.1 with an index value of 2.0.

### CORASON Phylogenetic Reconstruction of BGCs

The phylogenic reconstruction of four types of BGCs [aryl polyene, NRP, siderophore, and PK type I (T1PK)] was analyzed with CORASON version 1.0 (Navarro-Munoz et al., 2020) and a query gene (Supplementary Figure 1). First, a database for each type of BGCs was created from all the BGCs of the same type recovered in the genomes and metagenomes, which were obtained from our previous antiSMASH version 5.0 analysis. Then a BGC from each of our databases was chosen as the reference, and a gene from the BGC core was chosen as the query protein. A BGC core represents all the genes which encode a biosynthetic enzyme involved in a metabolic transformation during the biosynthesis. In additional to the BGC core, BGC contains other genes which may not be required for biosynthetic transformation but play important roles in the BGC, such as transport-related genes or regulatory genes. The choice of the reference BGC and query protein for a database was made by taking different variables into account: (1) the reference BGC must be one of the longest BGCs in the database, (2) the query protein must come from a biosynthetic core gene or an additional biosynthetic gene close to the core, and (3) the query protein must be present in at least half of the BGCs in the database.

Once a reference BGC and a protein query were selected for a database, CORASON version 1.0 was used to determine the core of this type of BGCs and to infer the phylogenies. For the phylogenetic reconstruction, the core BGCs are concatenated and aligned using Muscle version 3.8.31 (Edgar, 2004), and the phylogeny is inferred using the maximum likelihood method in FastTree version 2.1.10 (Price et al., 2009). Specific details for the method are available in Navarro-Munoz et al. (2020). If less than half of the BGCs present in a database appear in the phylogeny or if many BGCs appear more than once in the phylogeny (which may happen if the selected query protein belongs to a biosynthetic gene that can be found multiple times in the same BGC), the reference BGC and query protein previously selected are deemed to be inadequate for the analysis. In this case, the process to choose a reference BGC and query protein was done once more, following the same procedure as before. The reference BGC and query protein selected for the four types of BGCs studied are presented in Supplementary Table 8.

## Results

### Mining Bacterial Genomes

The bacterial genomes (*N* = 14) studied herein originated from cultured gut bacteria associated with *C. varians* and *C. rohweri*. Among these 14 bacterial genomes, only three genomes, all from *C. varians*, did not possess any BGC: Burkholderiales sp. Cv33a, *Cephaloticoccus capnophilus* Cv41, and Rhizobiales sp. JR021-5. In the other bacterial genomes, a total of 31 BGCs have been found, and the type and length of the 31 BGCs are listed in Supplementary Table 4. In bacteria associated with *C. varians*, 10 BGCs were found representing seven types of BGCs: aryl polyene, beta-lactone, ectoine, NRP, resorcinol, T1PK, and terpene. In bacteria associated with *C. rohweri*, 21 BGCs were found representing six types of BGCs: aryl polyene, beta-lactone, ladderane, NRP, siderophore, and T1PK. In *C. varians*, the majority of BGCs originate from Burkholderiales symbionts, whereas in *C. rohweri*, they originate from different bacterial orders (Burkholderiales, Pseudomonadales, and Xanthomonadales) (Supplementary Figure 2).

### Assessing the Quality of Metagenomic Data

The analysis of the 17 *Cephalotes* metagenomes resulted in 168 bins. Almost half the bins (72 out of 168) have a predicted completeness higher than 90%, 48 bins have a completeness higher than 70%, 31 bins have a completeness higher than 50%, and only 17 bins have a completeness lower than 50% (Supplementary Table 3). Concerning the contamination, half the bins (85 out of 168) have a predicted low contamination level (<5%). The 168 bins were identified from 31 different bacterial orders (Supplementary Figure 3A), and 54% of the bins were not contaminated with different bacterial orders (Supplementary Figure 3B). Mining these bins using antiSMASH version 5 (Blin et al., 2019) resulted in a total of 233 BGCs found in 111 bins (Supplementary Table 5). The BGCs were identified from 21 different bacterial orders. The bacterial orders having the highest number of BGCs are Burkholderiales (94 BGCs), Pseudomonadales (42 BGCs), Xanthomonadales (24 BGCs), and Rhizobiales (19 BGCs).

To further evaluate the quality of the metagenomic data, we investigate the genomic similarity of each metagenomic bin to the cultured isolate genomes from *C. rohweri* and *C. varians* (seven strains from each ant species). We created a heatmap representing the gANI and the AF values as a proxy of the sequence similarity (Supplementary Figure 4 and Supplementary Table 6; Altamia et al., 2020). The mapping of bins to cultured isolate genomes was considered successful if it met the gANI × AF > 80 threshold. In *C. rohweri*, six out of 11 metagenomic bins were successfully mapped at the species level (gANI > 0.95) to seven cultured isolate genomes (Supplementary Figure 4A). Metagenomic bins 5 and 12 were mapped to three *Ventosimonas* sp. genomes, bin 9 was mapped to the genome of Xanthomonadales sp., bins 14 and 15 were mapped to three Burkholderiales sp. genomes, and bin 16 was mapped to the genome of *Cephaloticoccus primus*. In *C. varians* PL005W, four out of eight metagenomic bins were successfully mapped at the species level (gANI > 0.95) to four genomes (Supplementary Figure 4B). Metagenomic bin 4 was mapped to the genome of *Ventosimonas gracilis*, bins 7 and 8 were mapped to Burkholderiales sp. genomes, and finally bin 10 was mapped to the genome of *C. capnophilus*.

We compare the number of shared BGCs between metagenomic bins and genomes (Supplementary Figure 5 and Supplementary Table 7). In *C. rohweri*, 11 BGCs were shared including six from the bacterial order Burkholderiales (two NRPs, two beta-lactones, and two T1PKs), four from Pseudomonadales (two aryl polyenes and two NRPs), and one from Xanthomonadales (one ladderane). The analysis shows that 10 BGCs were unique to the genomes and that 23 BGCs were unique to the metagenomes. In *C. varians* PL005W, two BGCs were shared: one T1PK from Burkholderiales and one aryl polyene from Pseudomonadales. Additionally, six BGCs were found only in the genomes, and 16 BGCs were found only in the metagenomes.

In the *C. varians* PL010W metagenome, only one bin was mapped to a Burkholderiales sp. genome with the gANI × AF > 80 threshold (Supplementary Figure 4B). Four BGCs were identified (Supplementary Table 5), and none of them matched any BGCs found in the *C. varians* genome (Supplementary Table 7). Because of the low numbers of BGCs, we removed this metagenome for further analysis and used only the *C. varians* PL005W metagenome for this species.

### Diversity of BGCs Across the *Cephalotes* Phylogeny

From the 233 BGCs retrieved in the genomes and metagenomes, we identified 19 different types of BGCs: acyl amino acids, aryl polyene, bacteriocin, beta-lactone, butyrolactone, cyclodipeptide (CDP), ectoine, furan, hserlactone, ladderane, lanthipeptide, linear azol(in)e-containing peptides (LAPs), NRP, phenazine, resorcinol, siderophore, T1PK, terpene, and thiopeptide (Supplementary Table 5). The aryl polyene and NRP BGCs were the most numerous with 66 and 57, respectively, retrieved in the genomes and metagenomes. The type and length of all the BGCs are presented in Supplementary Tables 4, 5. Few bacterial BGCs found in the metagenomic analysis are hybrids made of different types of BGCs: aryl polyene–beta-lactone–resorcinol, aryl polyene–ladderane, aryl polyene–resorcinol, and NRP–T1PK.

**FIGURE 1:**
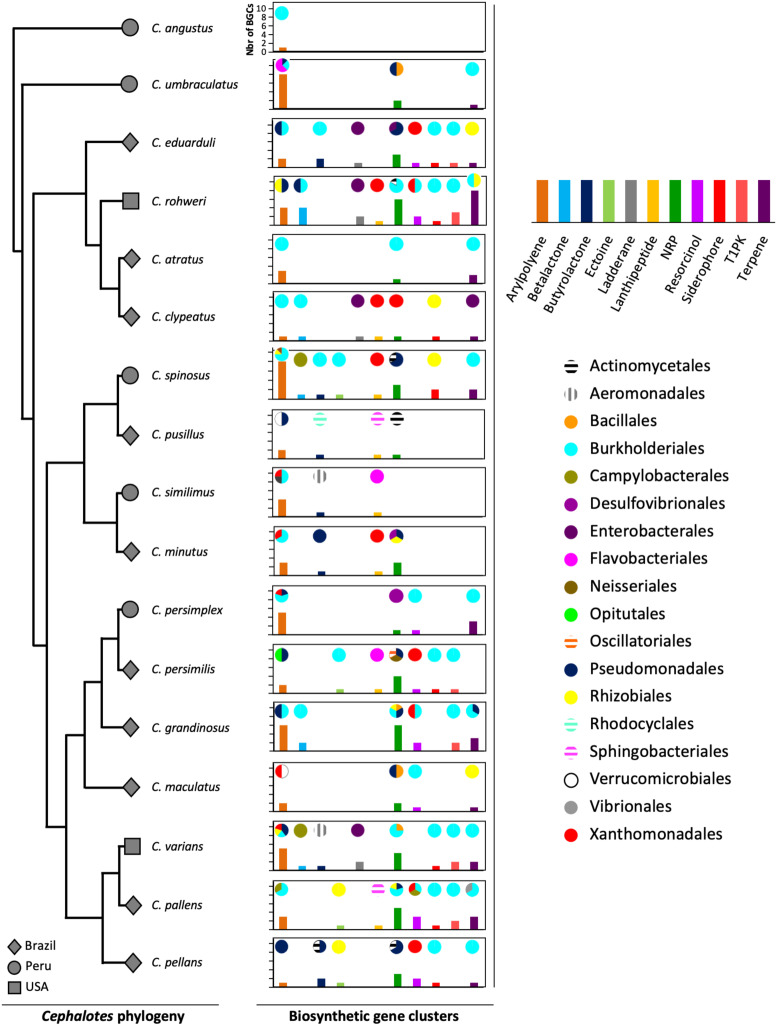
Diversity of BGCs of bacteria associated with *Cephalotes* turtle ants. The gray symbols represent the country from which an ant originated. The graphs represent the bacterial BGCs found in the metagenomes of each *Cephalotes* species in this study. The bars of the graphs represent the number of BGCs of each type found in the metagenomes. The pies represent the proportion of bacterial orders from which each BGC type originated.

The gut metagenomes of 17 species of *Cephalotes* studied here were collected in 3 different countries: 10 species from Brazil, 5 species from Peru, and 2 species from the United States. The lowest number of BGCs (*N* = 1) was found in *Cephalotes angustus*, the highest number of BGCs (*N* = 31) was recorded for *C. rohweri*, and a mean of ∼14 BGCs per species (Supplementary Table 5) was found. In Figure 1, the most abundant types of BGCs, those having at least five BGCs found in metagenomic bins, are shown across the *Cephalotes* phylogeny, representing a total of 202 BGCs originating from 19 different bacterial orders. The vast majority of BGCs were identified in Burkholderiales, Pseudomonadales, Xanthomonadales, and Rhizobiales.

### Sequence Similarity Networking and BGC Phylogenies

**FIGURE 2:**
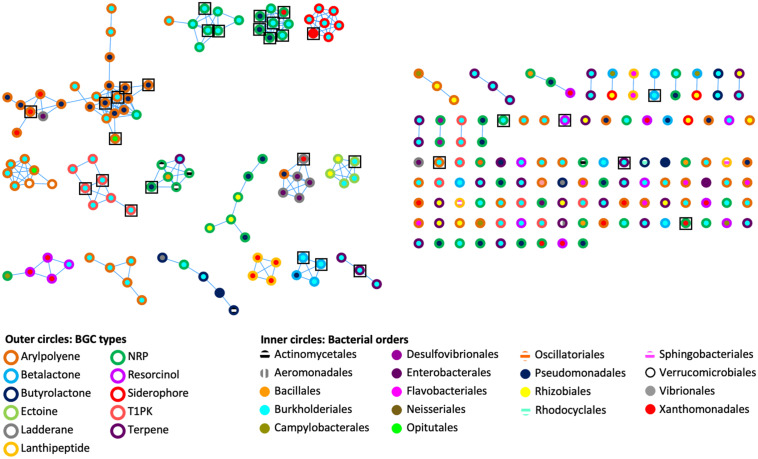
Genetic networks of the BGCs identified in bacterial genomes and *Cephalotes* gut metagenomes. A distance threshold of 0.45 and an MCL index value of 2.0 were applied to generate the network. Color codes are respective for bacterial BGC type and bacterial order. Black squares represent the 31 BGCs found in bacterial genomes.

To assess the genetic architectural diversity of BGCs (number and type of gene, gene order, gene domain, and BGC length), we used BIG-SCAPE version 1.0.0 (Navarro-Munoz et al., 2020) to create a sequence similarity network of the BGCs found in the genomes and metagenomes (Figure 2). We used the type of BGCs and the bacterial order to color the outer and inner circles of nodes, respectively. In this analysis, 31 networks of BGCs are formed, each containing between 2 and 24 nodes, and 90 nodes are singletons. Each network is mainly composed of the same type of bacterial BGCs. A third of the networks contain one or two BGC nodes of a different type than the rest of the nodes composing this network. Additionally, in half of the networks, BGCs originated from the same bacterial order, while in the other half, the BGCs originated from two or three different bacterial orders. Notably, the 26 out of 31 BGCs from cultured isolate genomes are clustered in networks shared with BGCs from metagenomic bins.

**FIGURE 3:**
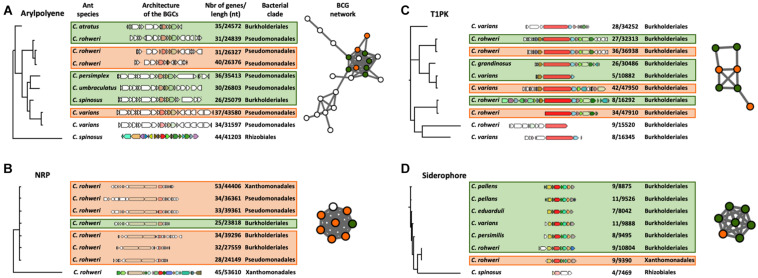
CORASON phylogenetic reconstruction of aryl polyene **(A)**, NRP **(B)**, T1PK **(C)**, and siderophore **(D)** BGCs. Because of their large size, only a selected clade of the aryl polyene and NRP phylogenies is presented here. The whole phylogenies of these clusters can be found in Supplementary Figures 6, 7. These phylogenies show the genomic composition of each BGC, the size and number of genes of each BGC, the bacterial origin of the BGC (on the right of the BGC), and the host ant species (on the left of the BGC). Each colored BGC in the phylogeny is represented as a node in the networks which were generated in the previous genetic network analysis (Figure 2). The nodes in the network are colored orange for BGCs originating from a bacterial genome or green for BGCs originating from a metagenome. The white nodes represent BGCs present in the network analysis but were not found in the phylogenetic clade presented here.

The phylogenetic reconstruction of the BGCs using a query gene was inferred using CORASON version 1.0 (Navarro-Munoz et al., 2020; Supplementary Figures 3, 6, 7 and Supplementary Table 8). Four types of BGCs, aryl polyene, NRP, siderophore, and T1PK, were chosen for this analysis since they are abundant in genomic data and also because these chemical families are thought to play an important role in bacterial colonization, interaction, and competitiveness (Zhang et al., 2009; Chaturvedi et al., 2012; Shevchuk et al., 2014; Schöner et al., 2016). With an aryl polyene KS gene as a query gene, 38 different BGCs were retrieved in the aryl polyene BGC phylogeny out of the 66 detected in the *Cephalotes* bacterial genomes and metagenomic bins (Supplementary Figure 6). These 38 aryl polyene BGCs form several distinct clades. One clade is composed of nine BGCs which are grouped into the largest aryl polyene network (Figure 3A). These BGCs originated from the genomes and metagenomic bins of Burkholderiales and Pseudomonadales isolated from six different ant species (Figure 3A). In this clade, the aryl polyene BGCs have a length of between 24,572 and 43,580 nucleotides (nt) and have 26–44 genes. In the NRP BGC phylogeny based on an AMP-binding gene (adenylation domain), 33 different NRPs were retrieved out of the 57 detected in the *Cephalotes* bacterial genomes and metagenomic bins (Supplementary Figure 7). These 33 NRP BGCs form several distinct clades including a polytomy composed of seven BGCs found in a network (Figure 3B). In this clade, the NRP BGCs were identified in the genomes and the metagenomic bins from three bacterial orders (Burkholderiales, Pseudomonadales, and Xanthomonadales) isolated from a single ant host (*C. rohweri*). NRP BGCs have a length of between 23,818 and 53,610 nt and have 25–53 genes in this clade. The PK synthase (PKS)–KS gene results in the T1PK BGC phylogeny of 10 BGCs out of the 15 detected in the *Cephalotes* bacterial genomes and metagenomic bins (Figure 3C). All BGCs originated from the genomes and metagenomic bins of Burkholderiales isolated from six different ant species. T1PK BGCs have a length of between 10,882 and 47,950 nt and have 5–42 genes. Seven T1PK BGCs were found in a single network cluster, and the three T1PK BGCs remaining all fall as singletons in the sequence similarity network analysis (Figure 2). The IucA–IucC gene was used to infer the siderophore BGC phylogeny. Eight siderophores were retrieved out of the 10 detected in the *Cephalotes* bacterial genomes and metagenomes (Figure 3D). Siderophore BGCs have a length of between 7,469 and 10,804 nt and have 4–11 genes. A network matches seven siderophore BGCs from the genomes and metagenomic bins of Burkholderiales and Xanthomonadales, each associated with a different ant species.

**FIGURE 4:**
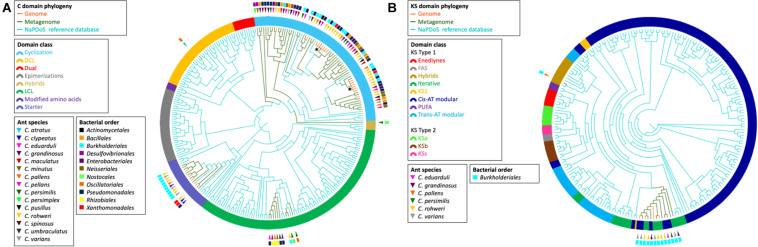
Phylogeny of retrieved C and KS elongation domain sequences in the NRP and PK assemblies, respectively. The phylogenies were inferred from the C domain sequences **(A)** and the KS domain sequences **(B)** from the *Cephalotes* bacterial genomes (orange branches) and metagenomes (green branches) with the NaPDos reference database (blue branches). The ring surrounding the tips represent the C domain class or KS domain class. The bacterial order from which each domain sequence was retrieved is indicated by the colored rectangle. The ant species associated with each sequence is indicated by the colored triangle. The symbol * designates the clade of the two C domain sequences found in the NRP phylogenetic reconstruction (see Figure 3).

The elongation domain phylogenies of NRP synthase and PK synthase (PKS), C and KS domains, respectively, were inferred using NaPDos (Ziemert et al., 2012) to check if the sequences of known metabolites matched our data (Figure 4). The majority of the identified C domain sequences (66 out of 89) from genomes and metagenomes belong to the cyclization domain class catalyzing both peptide bond formation and subsequent cyclization. The retrieved KS domain sequences (*N* = 13) from genomes and metagenomic bins fall into a single clade corresponding to the *Cis*-AT modular class or the iterative domain class. It has been shown that the C and KS domains are the most informative in predicting pathway associations of NRP and PK clusters, respectively (Rausch et al., 2007; Jenke-Kodama and Dittmann, 2009). Unfortunately, no close similarity with the identified C and KS sequences of known NRP and PK were found when comparing with NaPDos and the NCBI database; thus, no prediction could be made about the chemical structure of bacterial secondary metabolites.

## Discussion

Since the expansion of computational tools for mining microbial genomes, the discovery of BGCs of interest for pharmaceutical applications has become a growing field (Ziemert et al., 2016). This approach is complementary to natural products chemistry and metabolomics in helping with the prioritization of strains producing potential drugs with original chemical structures. Genome mining studies on prokaryote genomic data have revealed that BGCs are distributed in large gene cluster families, with the vast majority of them still being uncharacterized (Cimermancic et al., 2014). Some of these BGCs are distributed widely across the entire bacterial domain, with the most prominent being aryl polyenes, PKs, saccharides, and siderophores, having known and putative functions in microbe–host and microbe–microbe interactions (Cimermancic et al., 2014). The rise of advanced genomic tools to mine genomes and predict the chemical structure of metabolites from BGCs will certainly benefit the field of host–microbe symbiosis. Herein, we present the first genome and metagenome mining study of gut bacteria across different species of herbivorous turtle ants to assess the diversity of BGCs in the genome of nutritional symbionts. When applied across the host phylogeny, a genome mining approach provides crucial information about how the evolutionary history of the host may impact the diversity of BGCs in the symbiont genome. For instance, BGCs can undergo a vast array of evolutionary process through *de novo* assembly, gene duplication/deletion, and horizontal gene transfer, which drive microbial chemodiversity (Rokas et al., 2020). The systematic study of BGC diversity of symbionts across host species from different geographic areas may provide a list of BGCs likely to play a crucial functional role for the symbiont and for the host.

Recent studies mining metagenomes for BGCs include the defensive symbionts associated with the eggs of the beetle *Lagria villosa* (Waterworth et al., 2020), with the marine sponge *Mycale hentscheli* (Storey et al., 2020), and with the gill of shipworm (Altamia et al., 2020). In the latter study, the quality of the metagenomic data was evaluated by mapping the metagenomic bins to the culture isolate genomes to assess sequence similarity. In our case, the main limitation is the culture of *Cephalotes* gut symbionts, which are difficult to grow *in vitro*. To date, 14 strains have been isolated in *C. varians* and *C. rohweri*, and 10 out of 19 metagenomic bins from these two species were mapped to cultured isolate genomes (Supplementary Figure 4 and Supplementary Table 6). Although the gANI × AF values (from 0.76 to 0.91) indicate moderate to good sequence similarity between genomes and bins, the percentage of contigs mapped for each bin varies from 11 to 83%. Despite the fact that having more cultured isolate genomes could increase the mapping of bins to genomes, we cannot rule out partial misassembly of metagenomic bins. Notably, several BGCs detected in metagenomic bins present high sequence similarity and high percentage of contigs mapped with those retrieved from genomes, suggesting good confidence in the metagenome-assembled genome analysis (Supplementary Figure 5 and Supplementary Tables 7, 8). Although the contamination of bins can be high in some cases, here, we were looking for the highest bin completeness to assess the largest diversity of BGCs.

In the network analysis, 26 out of 31 BGCs from cultured isolate genomes were found in networks shared with BGCs retrieved from metagenomes (Figure 2). Additionally, some core gene sequences from genomic and metagenomic data of the BGCs of aryl polyene, NRP, T1PK, and siderophore fall into the same clade in the respective reconstructed phylogenies (Figure 3). It is interesting to note that the selected BGCs in Figure 3 reveal different patterns of distribution. For instance, one siderophore BGC shows high similarity in a single symbiont across different ant host species, whereas one BGC encoding the production of NRP found different symbionts within a single host species. These patterns could reflect different mechanisms of BGC conservation within symbionts and across host species. Future work will focus on understanding the evolutionary relationships of these BGCs. In parallel, the C and KS domain sequences from genomes and metagenomes are also found in the same phylogenetic clade (Figure 4). We do not know how much of the underlying BGC diversity is captured by these metagenomes, but taking together the strategy of mining genomes and metagenomes provides a complementary approach to explore the BGC diversity in insect–microbe mutualism, especially when gut symbionts are difficult to culture. More work in natural product chemistry is needed to characterize the specialized metabolites produced by these BGCs and to demonstrate their role in the multipartite mutualisms between gut symbionts and *Cephalotes* ants.

The low number of metagenomes (*N* = 17) collected in a large geographic range (Brazil, Peru, and the United States) prevents us from testing whether the host geography impacts the symbionts’ BGCs. More genomes and metagenomes covering a smaller geographic area are needed to test the geographic mosaic theory of coevolution, which stipules that in strongly interacting species, geography and community ecology can shape coevolution through local adaptation (Thompson, 2005; Nuismer, 2006). For instance, in the symbiosis between antibiotic-producing cuticular symbionts and fungus-growing ants, it was demonstrated that the geographical isolation of ant populations in Central America has an impact on the antibiotic potency of the locally adapted symbiont but not on the BGC architecture (Caldera et al., 2019). Further effort is required to test if this pattern applies to the conserved gut symbionts of *Cephalotes* turtle ants.

Four bacterial orders possess more than 80% of all the 233 BGCs detected in the *Cephalotes* bacterial genomes and metagenomic bins: Burkholderiales (44%), Pseudomonadales (15%), Xanthomonadales (15%), and Rhizobiales (9%) (Figure 1 and Supplementary Tables 4, 5). Interestingly, these four bacterial orders belong to the *Cephalotes* core bacterial community (Hu et al., 2018). The *Cephalotes* core bacterial community is maintained across the *Cephalotes* phylogeny and benefits these herbivorous ants with a low-nitrogen diet by recycling nitrogen from urea into essential and non-essential amino acids. In many insect nutritional symbioses, the endosymbiont resides in host cells or bacteriocytes and has a reduced genome with several copies of the functional genes responsible for the biosynthesis of amino acids and vitamins to enrich in nutrients the host diet. The genome reduction of gut symbionts in *Cephalotes* turtle ants has not been investigated yet; however, the mechanism for maintaining different communities originating from five bacterial orders must be more complex compared to other symbioses involving a single gut symbiont (bean bugs, Itoh et al., 2019) or bacteriocyte-dwelling endosymbionts (*Camponotus* ants, Gil et al., 2003; beetles, Anbutsu et al., 2017). The conserved bacterial symbionts in *Cephalotes* have redundancy in N-metabolism, and our results show that these bacterial symbionts possess the vast majority of the BGCs detected. These findings could support the idea that metabolic functions of a complex microbiome are often selected not solely for direct benefit of hosts but also for sustaining the gut community (Powell et al., 2016).

This hypothesis is in accordance with recent findings in a bean bug which needs to acquire a specific *Burkholderia* gut symbiont from the soil environment in every new generation (Itoh et al., 2019). In this study, authors demonstrated that a large number of *Burkholderia* species were able to access the bean bug gut, but in experiments of co-inoculations, the specific *Burkholderia* symbiont always outcompetes all the other *Burkholderia* species. These findings highlight that *Burkholderia* gut symbionts colonize with high efficiency the insect gut, and this trait is associated with the production of amino acids and siderophores (Powell et al., 2016). The *Cephalotes* core symbionts are transmitted through oral–anal trophallaxis, and this behavior is prevalent in *Cephalotes* (Hölldobler and Wilson, 1990). Newly enclosed workers from *C. rohweri* performed oral–anal trophallaxis with mature adults prior the development of the proventricular filter, which later blocks the entry of transient bacteria (Lanan et al., 2016). This remarkable transmission mechanism ensures the maintenance of the core gut symbionts over evolutionary time, suggesting the maintenance of genes promoting gut colonization and bacterial interaction. The gut symbiont communities of *Cephalotes* originating from five bacterial orders need to colonize gut tissue, tolerate each other, and possibly participate in cross-feeding. These interactions between co-occurring gut symbionts may be through the production of specialized metabolites.

To identify the structural similarities of conserved bacterial BGCs across different hosts, we performed CORASON version 1.0 gene analysis on four types of BGCs: aryl polyene, NRP, T1PK, and siderophore (Figure 3 and Supplementary Figures 6, 7). The selected clades presented in Figures 3A,B show similarities in the sequence of the core genes for aryl polyene BGCs originating from Burkholderiales and Pseudomonadales and for NRP BGCs originating from Burkholderiales, Pseudomonadales, and Xanthomonadales. These aryl polyene and NRP BGCs are each clustered in the networking analysis (Figures 2, 3), demonstrating the architectural similarities. In the NRP BGC clade of Figure 3B, the query gene AMP binding (adenylation domain) and the two genes coding for a C domain have 100% similarity and 100% coverage across the BGCs retrieved from the six genomes and metagenomes of Burkholderiales, Pseudomonadales, and Xanthomonadales from the same host *C. rohweri*. This high similarity between the six C domain sequences from three bacterial orders is also confirmed in the phylogenetic analysis (Figure 4). This pattern suggests a possible convergence of the gene possibly *via* horizontal gene transfer in the gut of *C. rohweri*. In insect–bacteria symbiosis, horizontal gene transfer has been shown to occur even in bacteria going through genome reduction (Waterworth et al., 2020). However, the three sequences did not match any known sequence in the database, limiting the identification of the bacterial origin of these genes. On the other hand, the T1PK and siderophore core gene phylogenies (Figures 3B,C) show that BGCs from Burkholderiales across different host species share a strong likely genetic conservation. Several identical genes of T1PK and siderophore BGCs are present across the core gene phylogenies, supporting a conserved architecture which suggests a common origin or convergent evolution of Burkholderiales BGCs across *Cephalotes* species. Symbiotic convergent evolution has been reported in insect–bacteria symbiosis (López-Sánchez et al., 2009; McCutcheon and Moran, 2010), showing that insect symbionts retain the ability to produce a certain number of proteins and functions which have an importance for both bacterial and host survival. Further work focusing on the occurrence of structurally similar BGCs in the genomes of environmental bacteria of the same order could reveal the origin and evolution of the BGCs in the ant–bacteria mutualism.

A final remark concerns the division of labor in multipartite mutualism. In honey bees, the gut microbiota is dominated by five coevolved bacterial clusters. However, the gut bacterial species have different abilities in digesting polysaccharides, which can be explained by a divergence into different ecological niches inside the gut of their host (Zheng et al., 2019). In *Cephalotes* gut, four of the five conserved symbionts possess the majority of the retrieved BGCs, but Opitutales, a major player in the *Cephalotes* nutritional symbiosis, is mostly lacking BGCs. Although multiple bins of Opitutales were retrieved, only two BGCs (aryl polyene) were detected (Supplementary Figure 8). Opitutales are localized in the midgut, whereas Burkholderiales, Pseudomonadales, Rhizobiales, and Xanthomonadales are found in the ileum of the hindgut (Flynn et al., 2021). This spatial isolation prevents direct interaction of Opitutales with other symbionts. This result supports the idea that BGCs have an essential role for symbionts living in close proximity, and conversely, alone-living symbiont may lose BGCs encoding specialized metabolites during genome reduction.

Taken together, our results address the diversity of bacterial BGC in an ant–bacteria nutritional symbiosis. In contrast to symbionts undergoing genome reduction, which keeps only the gene involved in supplementing the host nutrition, it is possible that a broader gene selection occurs in the genomes of gut bacteria involved in multipartite mutualisms. Future works will focus on the functional role of the specialized metabolites involved in the ant–gut bacteria symbiosis to test their contribution to the symbionts’ maintenance.

## Data Availability Statement

The datasets presented in this study can be found in online repositories. The names of the repository/repositories and accession number(s) can be found below: https://datadryad. org/stash/share/ad5ECcQθnRPTG8RTEtMdfayθmrnkqrRcUldlI BynE-4.

## Author Contributions

AC, CM, and CD conceived the study, interpreted the results, and revised the manuscript. AC performed the analyses and drafted the manuscript. All authors contributed to the article and approved the submitted version.

## Conflict of Interest

The authors declare that the research was conducted in the absence of any commercial or financial relationships that could be construed as a potential conflict of interest.
