# Supplementary material for: Assessing Biosynthetic Gene Cluster Diversity of Specialized Metabolites in the Conserved Gut Symbionts of Herbivorous Turtle Ants
Source: Front Microbiol. 2021 Jun 29;12:678100. doi: 10.3389/fmicb.2021.678100 (PMC8277422; doi:10.3389/fmicb.2021.678100)
Supplement: Supplementary file 1 [file Data_Sheet_1.PDF]

Supplementary Material

Assessing biosynthetic gene cluster diversity of specialized metabolites in the conserved gut symbionts of herbivorous turtle ants

Anaïs Chanson[1], Corrie S. Moreau[2], Christophe Duplais[3*]

[1]Université de Guyane, UMR8172 Ecofog, AgroParisTech, CNRS, Cirad, INRAE, Université des Antilles, Kourou, France
[2]Departments of Entomology and Ecology & Evolutionary Biology, Cornell University, Ithaca, NY, USA
[3]CNRS, UMR8172 Ecofog, AgroParisTech, Cirad, INRAE, Université des Antilles, Université de Guyane, Kourou, France

Table of contents

Supplementary Figure 1. Summary of the *Cephalotes* genomes and metagenomes workflow. 2

Supplementary Figure 2. Assessment of the differences between the BGCs in the genome analysis. 3

Supplementary Figure 3. Assessment of the bacterial composition of the metagenomic bins. 4

Supplementary Figure 4. Heat map of relationships between genomes of isolated strains and metagenomic bins for *C. rohweri* (A) and *C. varians* (B). 5

Supplementary Figure 5. Number of shared BGCs found in the metagenomic bins and cultured isolate genomes, for *C. rohweri* and *C. varians*.. 6

Supplementary Figure 6. CORASON phylogenetic reconstruction the arylpolyene BGCs from the *Cephalotes* bacterial genomes and metagenomic bins. 7

Supplementary Figure 7. CORASON phylogenetic reconstruction of the NRP BGCs from the *Cephalotes* bacterial genomes and metagenomes. 8

Supplementary Figure 8. Number and type of BGCs identified in the bacterial genomes and metagenomic bins in the *Cephalotes* gut core bacterium. 9

Supplementary Table 1. Assembly statistics of genomes. 10

Supplementary Table 2. Assembly statistics of metagenomes.. 12

Supplementary Table 3. Statistical assessment of the metagenomic bins... 14

Supplementary Table 4. BGCs types identified in the bacterial genomes. 18

Supplementary Table 5. BGCs types identified in the metagenomic bins. 19

Supplementary Table 6. Genomic similarity measurement between metagenomic bins and cultured isolate genomes. 28

Supplementary Table 7. Genomic similarity measurement between BGCs from cultured isolate bacterial genomes and metagenomic bins. 34

Supplementary Table 8. Reference BGC and query protein selected in the CORASON phylogenetic analysis. ... 39

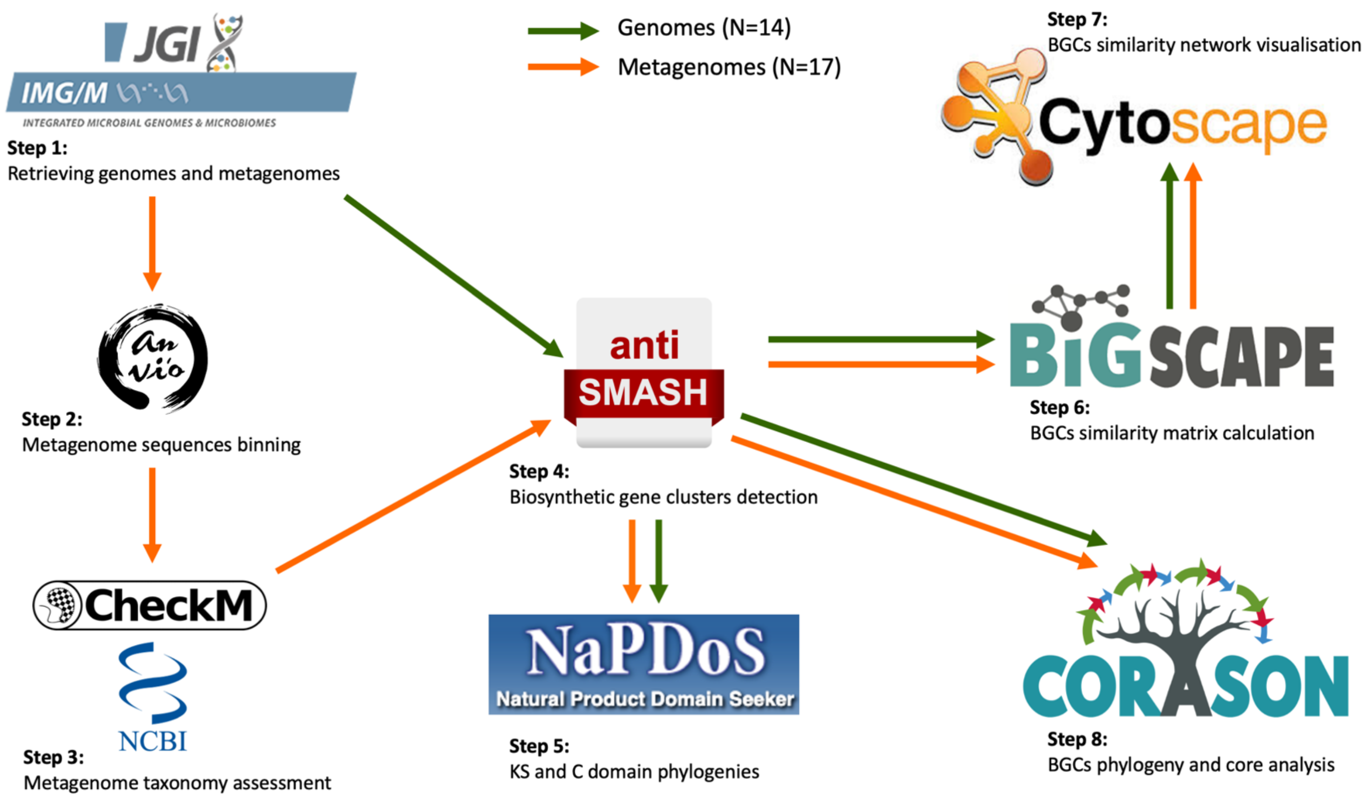

Supplementary Figure 1. Summary of the *Cephalotes* genomes and metagenomes workflow.

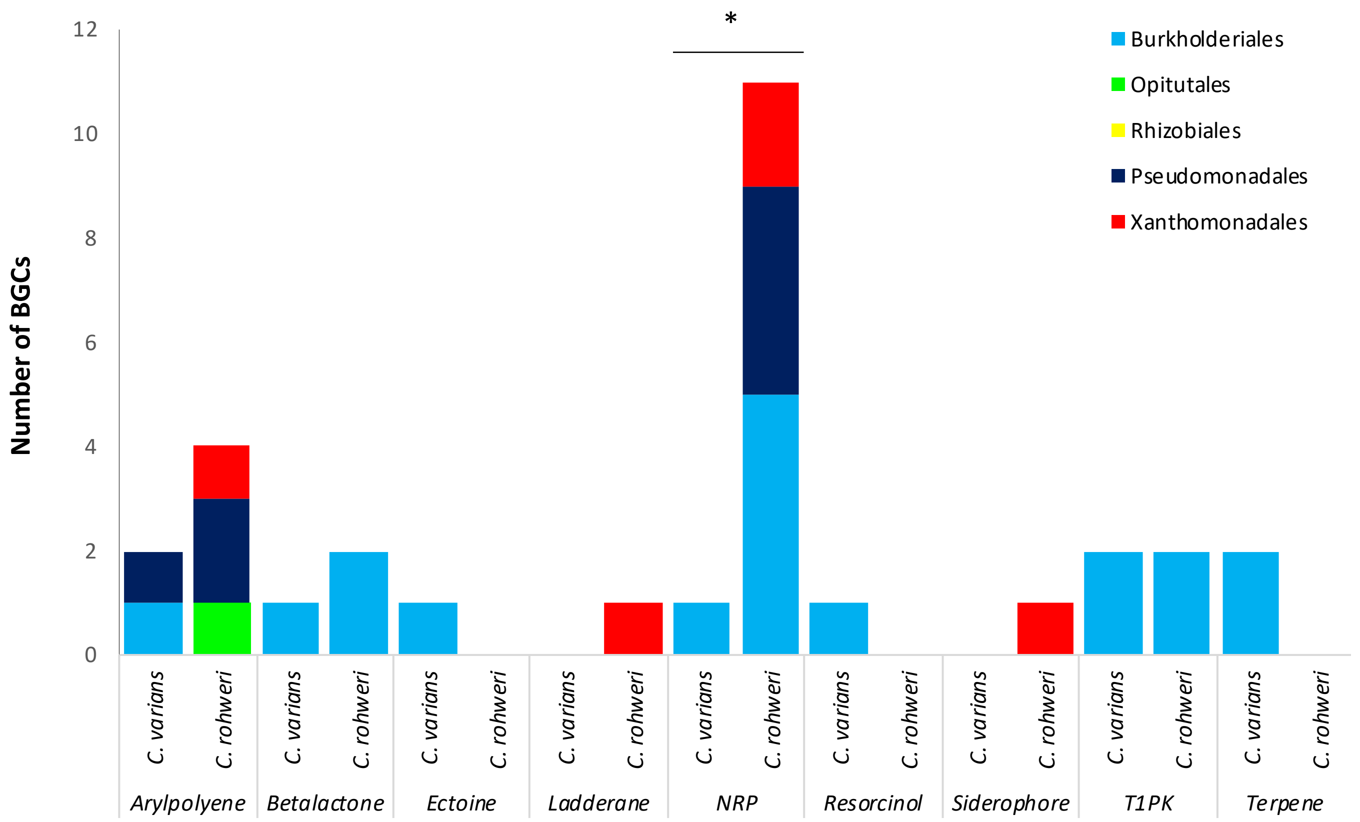

Supplementary Figure 2. Assessment of the differences between the BGCs in the genome analysis. Pairwise PERMANOVA analysis (999 permutations with false discovery rates correction) showing the statistical differences between the bacterial BGCs across isolated bacteria species and *Cephalotes* species. The symbol * represents a p-value lower than 0.05.

A

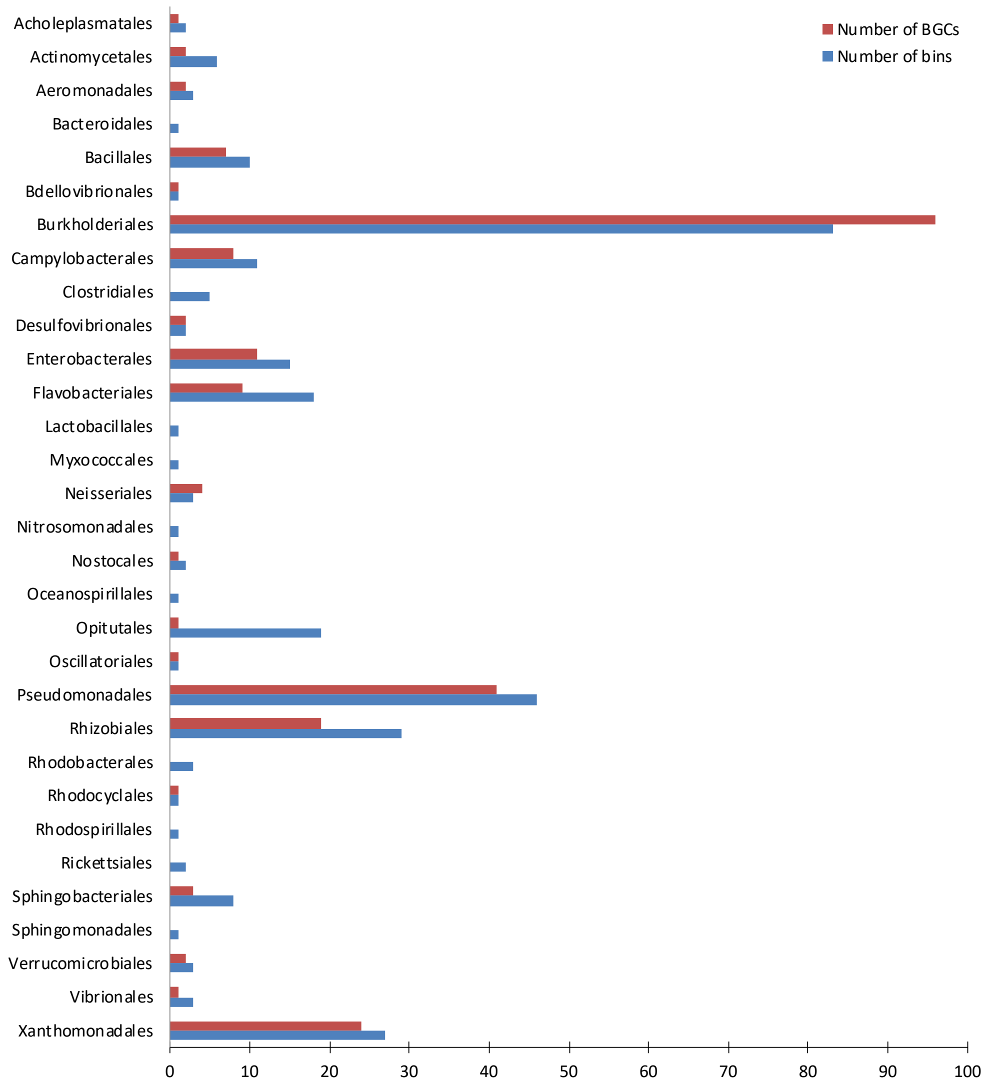

B

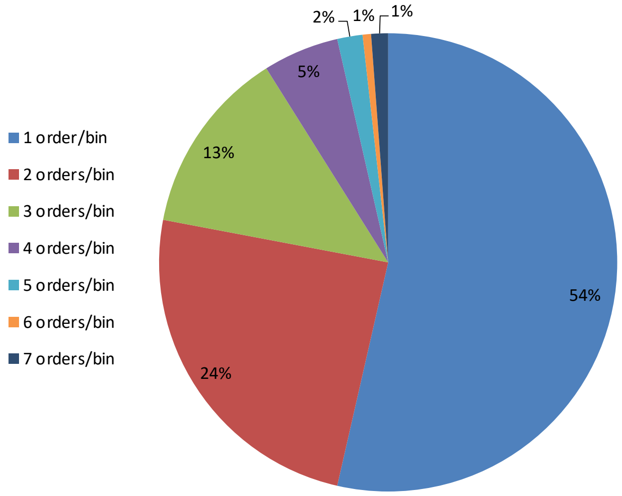

Supplementary Figure 3. Assessment of the bacterial composition of the metagenomic bins.
(A) List of bacterial orders identified in the bins identified in metagenomes. The blue bars represent the number of bins in which each bacterial order was found. The red bars represent the number of BGCs possessed by each bacterial order. (B) The proportion of different bacterial orders found in the bins.

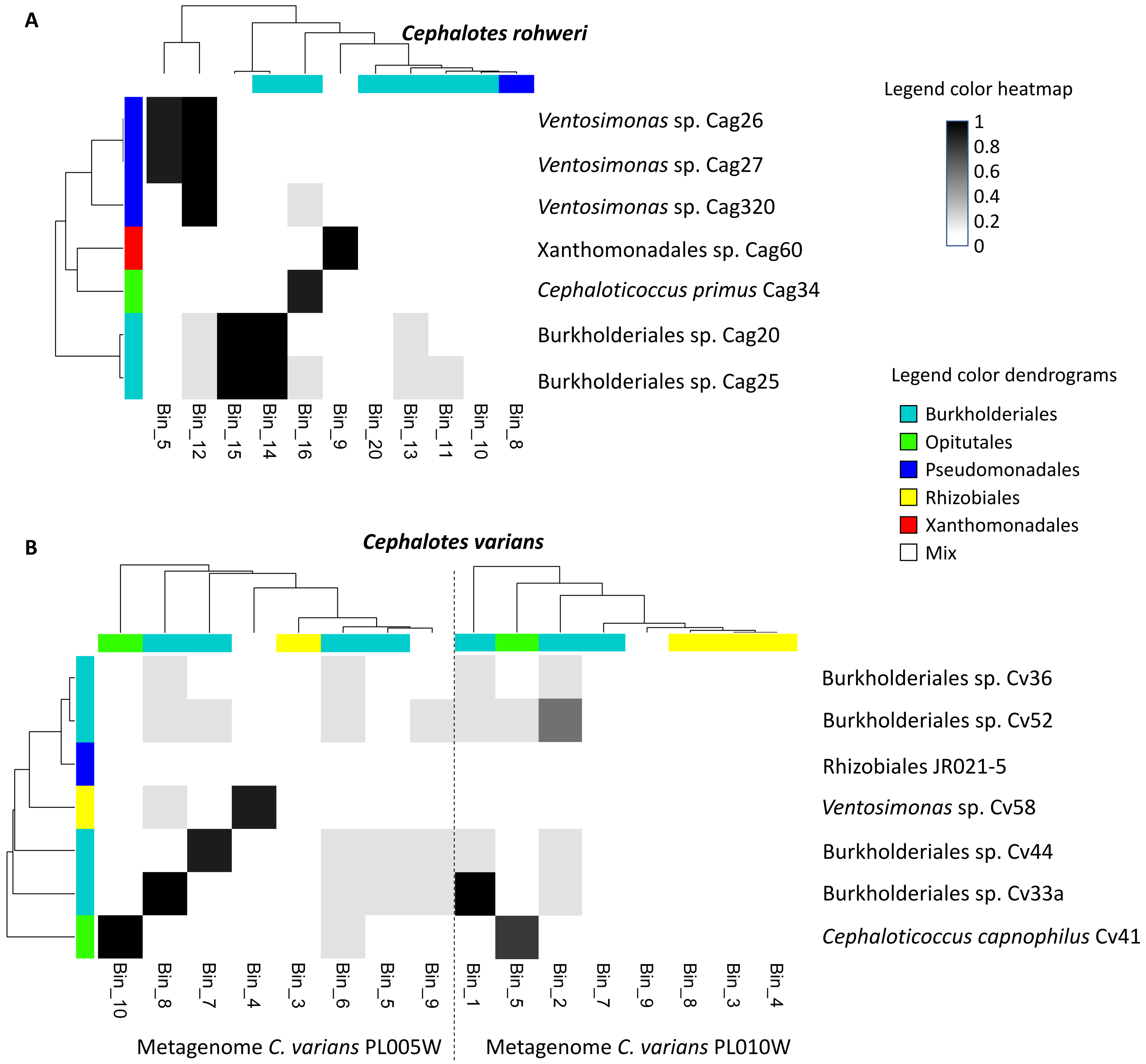

Supplementary Figure 4. Heat map of relationships between genomes of isolated strains and metagenomic bins for *C. rohweri* (A) and *C. varians* (B).

The scale bar of the heatmap is shaded from black to white according to gANI×AF calculated values. Color bars in the dendrograms indicate bacterial orders identified either in the metagenomic bins or in the cultured isolate genomes. In the color dendrograms legend 'mix' refers to metagenomic bins in which more than one different bacterial order was identified.

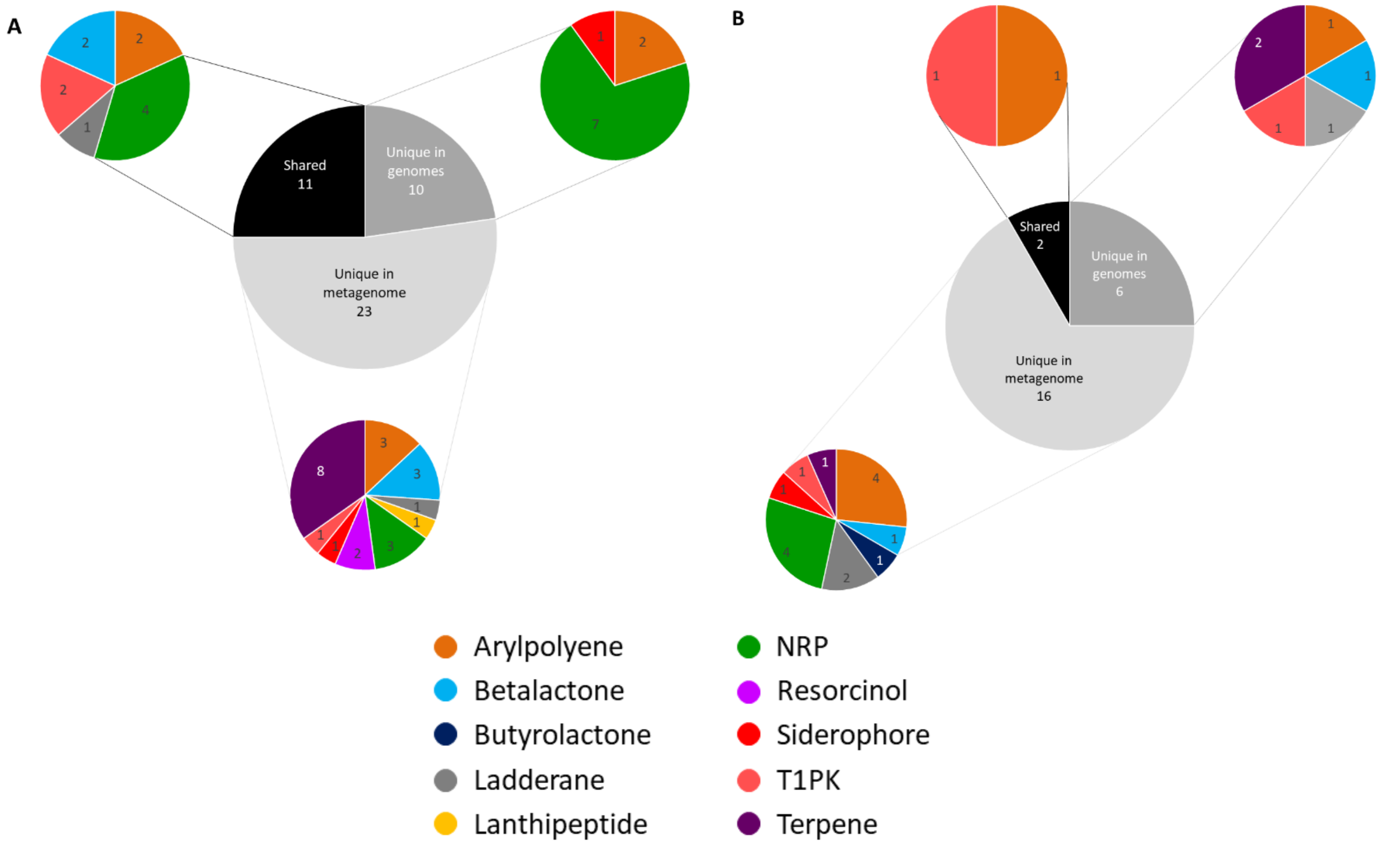

Supplementary Figure 5. Number of shared BGCs found in the metagenomic bins and cultured isolate genomes, for *C. rohweri* and *C. varians*.

The central pies represent the number of BGCs shared between the genomes and the metagenomes, and the number of BGCs which were only found in the genomes or only in the metagenomes. The peripheric pies represent the type and number of BGCs for each category (shared, unique in genomes or unique in metagenome). The color corresponding to each type of BGCs can be found in the legend.

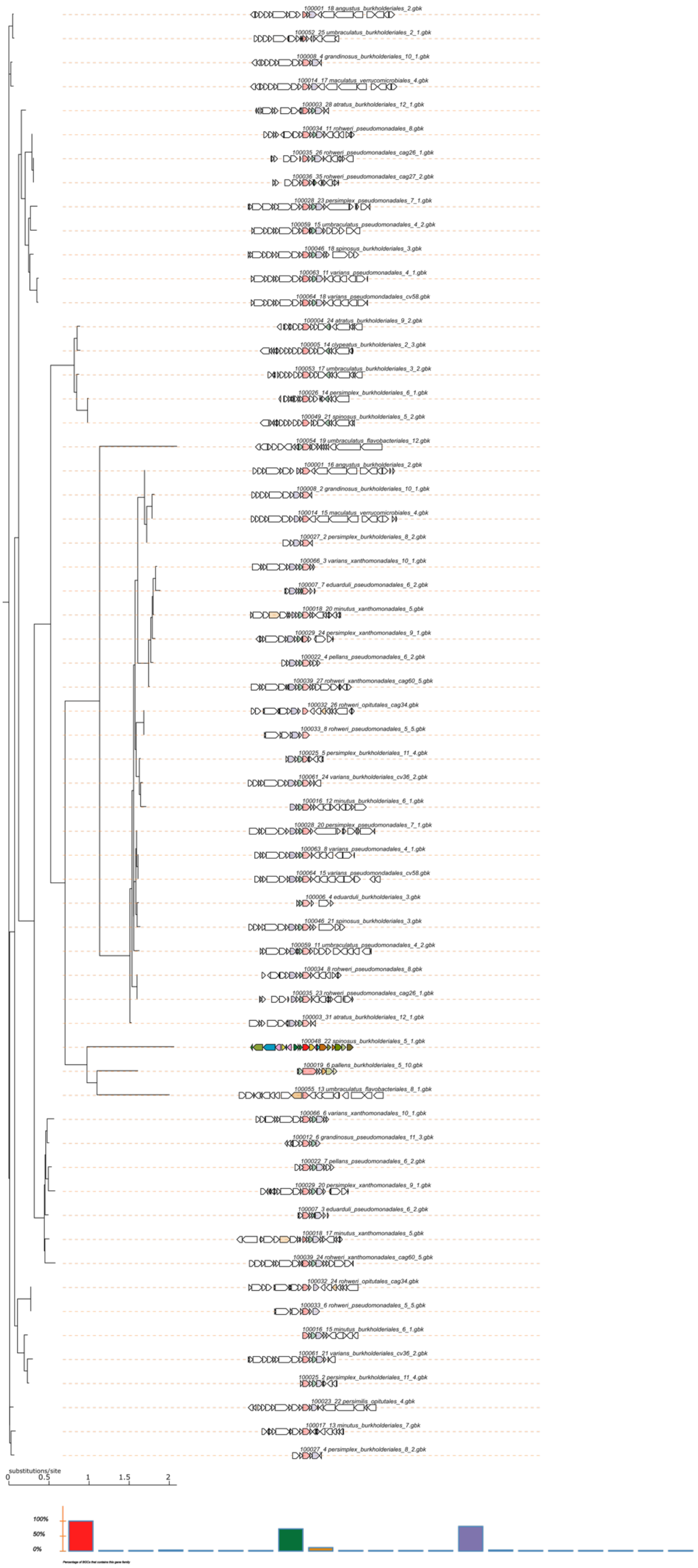

Supplementary Figure 6. CORASON phylogenetic reconstruction the arylpolyene BGCs from the *Cephalotes* bacterial genomes and metagenomic bins.

This phylogeny was implemented through CORASON by uploading all the arylpolyene BCGs retrieved from the *Cephalotes* associated bacterial genomes and metagenomic bins into a single database and by comparing their sequences and architectures to a reference BGC and query protein as detailed in Table S8. The histogram represents the percentage of BGC that contains this gene family.

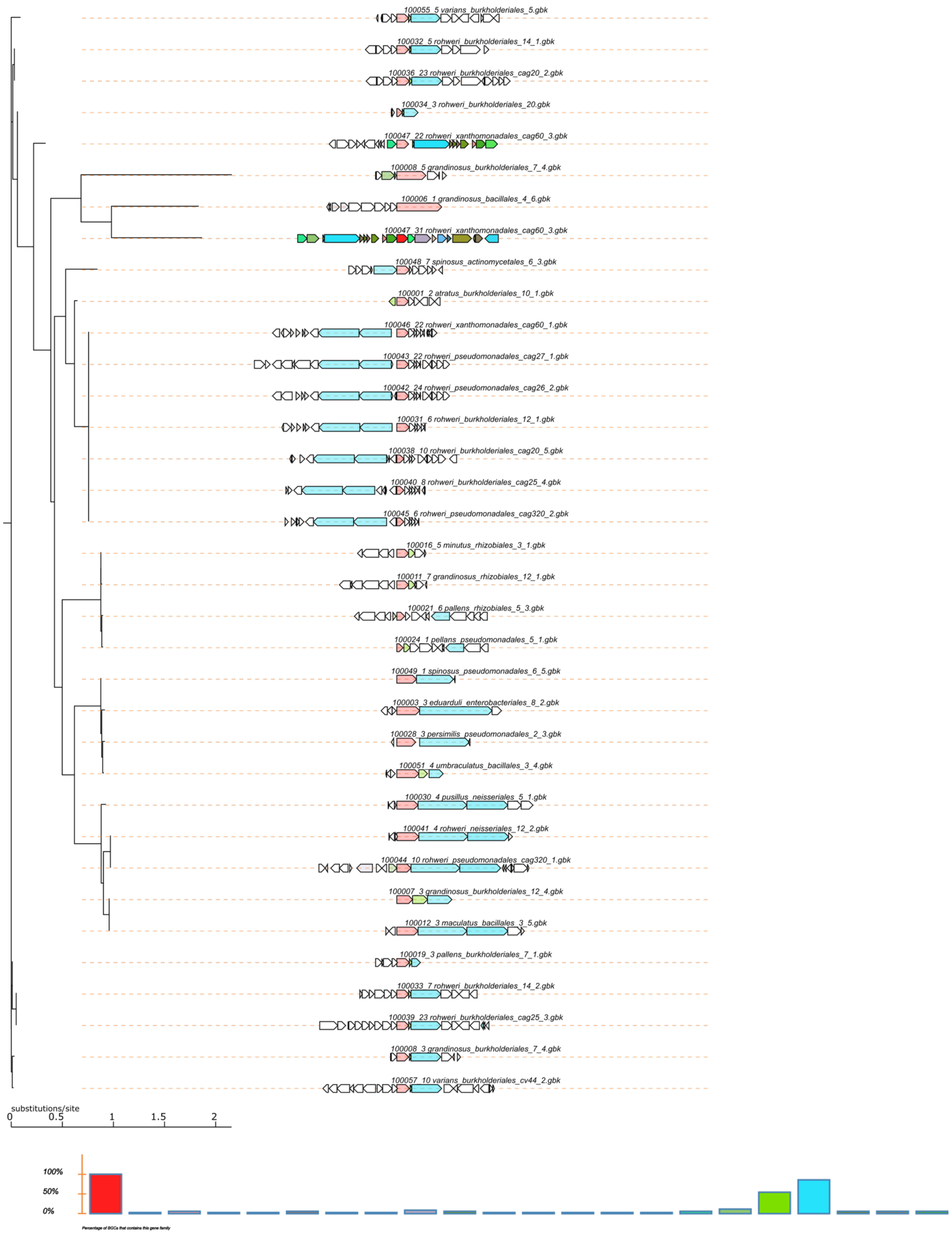

Supplementary Figure 7. CORASON phylogenetic reconstruction of the NRP BGCs from the *Cephalotes* bacterial genomes and metagenomes.

This phylogeny was implemented through CORASON by uploading all the NRP BCGs retrieved from the *Cephalotes* associated bacterial genomes and metagenomic bins into a single database and comparing their sequences and architectures to a reference BGC and query protein as detailed in Table S8. The histogram represents the percentage of BGC that contains this gene family.

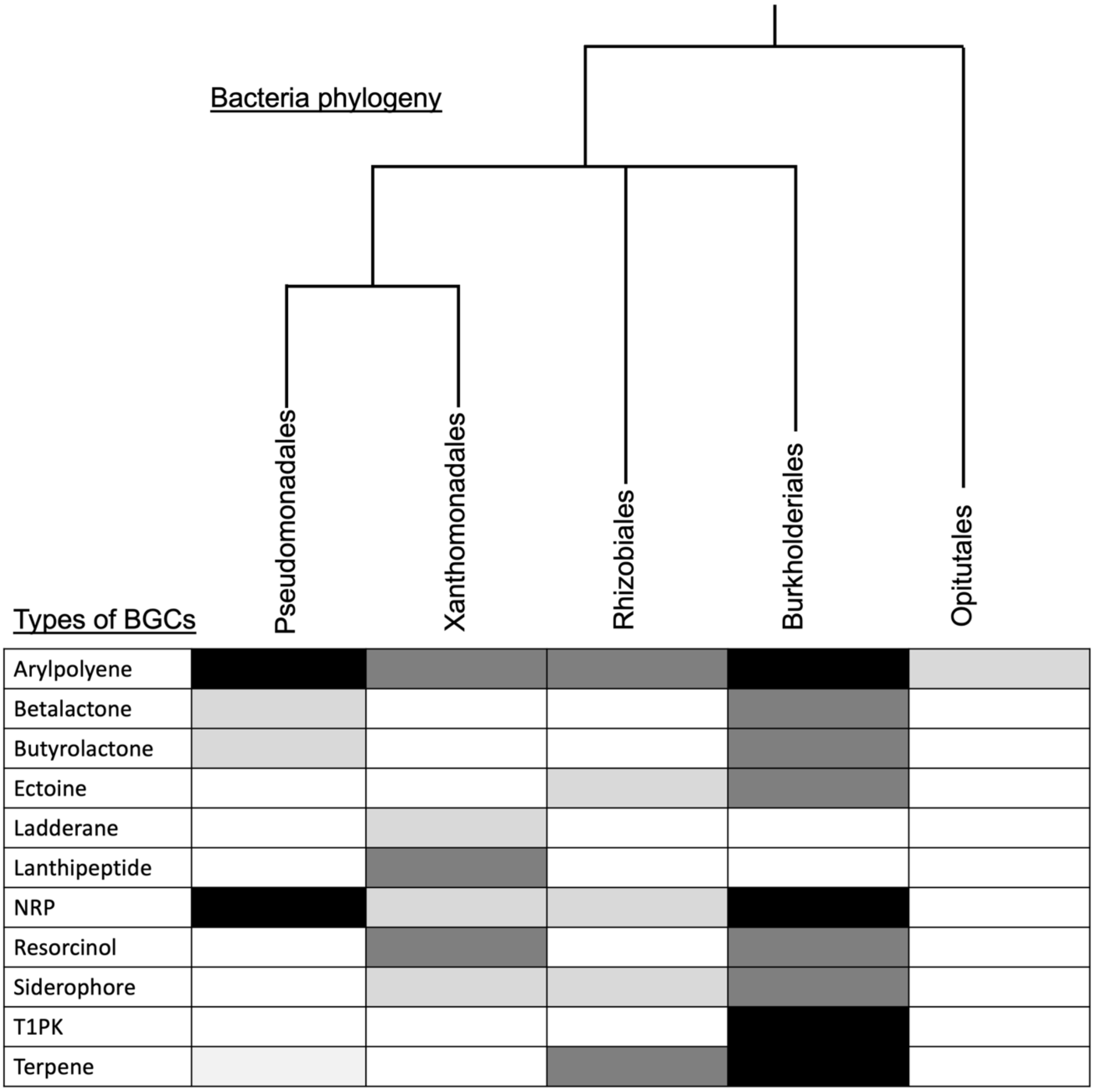

	Pseudomonadales	Xanthomonadales	Rhizobiales	Burkholderiales	Opitutales
Number of BGCs	48	28	19	115	2
Number of bins	53	32	30	105	21

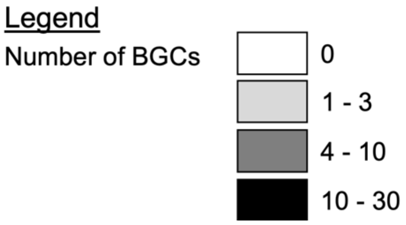

Supplementary Figure 8. Number and type of BGCs identified in the bacterial genomes and metagenomic bins in the *Cephalotes* gut core bacterium.

The black to white shading in the table represent the number of BGCs of each type identified in the genomes and metagenomes of the five main bacterial orders associated with the *Cephalotes* gut bacteria. The black to white shading represent the number of bins in which each bacterial order has been found.

Supplementary Table 1. Assembly statistics of genomes.
This table was extracted from the Supplementary Information of Hu *et al*., 2018.

Genome name	Taxonomic affiliation	Total length (bp)	Scaffold number	N50 (bp)	GC% mean	IMG project ID	Ant host
Cephaloticoccus primus Cag34	Order: Opitutales Class: Opitutae Phylum: Verrucomicrobia	2,353,466	23	207,028	62.43%	Gp0154034	*C. rohweri*
Cephaloticoccus canophilus Cv41	Order: Opitutales Class: Opitutae Phylum: Verrucomicrobia	2,094,663	34	169,871	59.29%	Gp0110136	*C. varians*
Rhizobiales sp. JR021-5	Order: Rhizobiales Class: Alphaproteobacteria Phylum: Proteobacteria	1,943,462	1	1,943,462	57.28%	Gp0155340	*C. varians*
Xanthomonadales sp. Cag60	Order: Xanthomonadales Class: Gammaproteobacteria Phylum: Proteobacteria	3,424,135	179	44,406	56.17%	Gp0127963	*C. rohweri*
Ventosimonas gracilis Cv58	Order: Pseudomonadales Class: Gammaproteobacteria Phylum: Proteobacteria	2,623,100	221	58,779	53.40%	Gp0110146	*C. varians*
Ventosimonas sp. Cag26	Order: Pseudomonadales Class: Gammaproteobacteria Phylum: Proteobacteria	2,729,219	92	79,155	54.82%	Gp0154031	*C. rohweri*
Ventosimonas sp. Cag27	Order: Pseudomonadales Class: Gammaproteobacteria Phylum: Proteobacteria	2,719,975	89	79,155	54.89%	Gp0154032	*C. rohweri*
Ventosimonas sp. Cag320	Order: Pseudomonadales Class: Gammaproteobacteria Phylum: Proteobacteria	2,825,500	94	108,424	55.67%	Gp0154033	*C. rohweri*
Burkholderiales sp. Cag20	Order: Burkholderiales Class: Betaproteobacteria	3,101,960	121	69,296	58.26%	Gp0154021	*C. rohweri*

	Phylum: Proteobacteria						
Burkholderiales sp. Cag25	Order: Burkholderiales Class: Betaproteobacteria Phylum: Proteobacteria	3,191,349	112	142,682	58.13%	Gp0154030	*C. rohweri*
Burkholderiales sp. Cv44	Order: Burkholderiales Class: Betaproteobacteria Phylum: Proteobacteria	3,159,338	601	33,742	60.40%	Gp0110143	*C. varians*
Burkholderiales sp. Cv33a	Order: Burkholderiales Class: Betaproteobacteria Phylum: Proteobacteria	2,469,676	150	48,592	58.91%	Gp0110144	*C. varians*
Burkholderiales sp. Cv26	Order: Burkholderiales Class: Betaproteobacteria Phylum: Proteobacteria	2,921,795	234	54,338	59.95%	Gp0110137	*C. varians*
Burkholderiales sp. Cv52	Order: Burkholderiales Class: Betaproteobacteria Phylum: Proteobacteria	2,935,707	203	93,689	60.36%	Gp0110145	*C. varians*

Supplementary Table 2. Assembly statistics of metagenomes.

Ant species	Bins number	Reads numbers	Total length Mbp	Total length Scaffold >=1Kbp (Mbp)	Scaffold number	Scaffold >=1Kbp number	N50 for scaffold length (bp)	GC% mean	Read coverage mean	IMG project ID
C. varians PL005W	11	40,804,316	184.98	142.43	281,884	129,829	1,074	39.18 %	16.60	Gp0095985
C. varians PL010W*	11	135,924,092	436.39	340.3	647,079	91,083	10,750	41.95 %	41.75	Gp009598
C. angustus	2	3,534,316	21.5	8.22	30,858	3,531	1,118	50.80 %	6.79	Gp0125961
C. atratus	15	34,205,408	218.88	141.37	198,281	67,767	1,488	38.53 %	9.20	Gp0125962
C. clypeatus	6	24,093,876	104.18	41.7	135,743	16,952	1,031	37.42 %	8.62	Gp0125963
C. eduarduli	8	22,505,270	82.63	37.38	100,267	12,793	1,259	39.99 %	9.53	Gp0125964
C. grandinosus	12	37,871,910	156.24	60.81	220,221	22,090	1,215	39.68 %	10.06	Gp0125967
C. maculatus	6	40,739,970	144.43	42.17	230,315	18,066	921	39.60 %	7.52	Gp0125968
C. minutus	8	31,576,424	115.64	37.69	180,629	15,101	1,038	38.38 %	8.10	Gp0125969
C. pallens	10	28,073,250	148.44	55.05	211,667	21,214	1,095	39.56 %	7.92	Gp0125970
C. pellans	7	33,836,572	142.82	43.13	224,021	18,268	934	38.32 %	8.51	Gp0126569
C. persimilis	6	28,044,242	106.24	44.84	133,984	19,037	1,077	40.41 %	10.09	Gp0126571
C. persimplex	11	40,440,804	231.49	102.43	291,903	53,617	1,090	37.94 %	10.14	Gp0126580
C. pusillus	5	18,252,188	61.89	24.03	93,263	9,713	1,389	42.35 %	6.67	Gp0126572
C. rohweri	22	58,943,942	305.84	271.58	142,961	78,950	3,982	40.47 %	22.40	Gp0126573
C. similimus	6	30,304,070	92.55	37.04	122,379	16,294	1,030	37.75 %	13.09	Gp0126574
C. spinosus	8	19,456,806	94.81	41	117,242	13,728	1,206	38.79 %	8.66	Gp0126575
C. umbraculatus	14	37,344,984	256.42	193.69	191,339	84,591	1,984	37.76 %	14.53	Gp0126577

*This metagenome was excluded in the final analysis
This table was extracted from the Supplementary Information of :
Hu, Y. *et al.* (2018) Herbivorous turtle ants obtain essential nutrients from a highly conserved nitrogen-recycling gut microbiome. *Nat. Commun.* 9, 964.

Supplementary Table 3. Statistical assessment of the metagenomic bins.

Host species	Number of bins		Completeness bin (%)	CheckM completness class	Contamination bin (%)	CheckM contamination class	Strain heterogeneity bin (%)	Number contigs bin	Number genes bin	Length bin (Mb)	Number of BGCs present
C. angustus	2	1	10.19	Partial	1.1	Low	0	316	1,506	1.23	0
		2	95.69	Near	0	Low	0	66	2,075	2.55	1
C. atratus	15	1	98.28	Near	2.77	Low	0	3,112	8,646	11.92	1
		2	96.55	Near	3.76	Low	0	2,760	8,783	10.34	1
		3	89.83	Substantial	28.68	Very high	31.94	323	2,509	2.22	0
		4	100	Near	0	Low	0	38	1,402	1.52	0
		5	100	Near	0	Low	0	176	2,053	2.21	0
		6	84.09	Substantial	15.99	Very high	0	729	5,094	4.73	0
		7	81.58	Substantial	2.07	Low	0	275	2,115	1.89	1
		8	96.55	Near	2.77	Low	0	85	2,053	2.14	0
		9	81.43	Substantial	25.49	Very high	65	455	3,733	3.5	2
		10	68.97	Moderate	31.56	Very high	4.44	910	4,862	4.23	2
		11	80.06	Substantial	32.37	Very high	38.46	798	4,765	4.39	2
		12	60.34	Moderate	21.22	Very high	18	523	2,575	2.21	1
		13	92.24	Near	0	Low	0	136	2,006	2.42	0
		14	82.52	Substantial	2.59	Low	0	425	2,743	2.77	0
		15	3.45	Partial	0	Low	0	194	423	0.542	0
C. clypeatus	6	1	98.58	Near	70.47	Very high	0	525	4,276	4.48	0
		2	94.83	Near	31.9	Very high	0	632	5,449	5.14	4
		3	99.5	Near	49.95	Very high	31.58	717	4,724	4.39	2
		4	98.28	Near	0	Low	0	143	1,824	2.03	0
		5	80.41	Substantial	1.72	Low	0	508	2,165	2.26	2
		6	98.28	Near	0.16	Low	0	805	3,940	4.53	0
C_eduarduli	8	1	96.55	Near	0	Low	0	448	2,497	2.8	1
		2	83.7	Substantial	0.86	Low	100	299	2,013	1.76	0
		3	98.28	Near	6.9	Medium	0	181	3,023	3.06	1
		4	86.41	Substantial	14.81	High	54.55	640	4,304	3.73	3
		5	98.28	Near	0	Low	0	71	2,152	2.61	0
		6	79	Substantial	27.9	Very high	52.63	588	4,057	3.9	3
		7	65.43	Moderate	20.85	Very high	53.33	526	2,793	2.28	0
		8	14.58	Partial	1.72	Low	100	736	4,462	3.88	6
C. grandinosus	12	1	98.28	Near	0.86	Low	100	79	1,817	1.99	0
		2	68.5	Moderate	0.31	Low	0	523	2,225	2.23	0
		3	96.55	Near	0	Low	0	90	2,357	2.46	1
		4	82.45	Substantial	24.48	Very high	10.53	871	5,889	5.54	8
		5	88.79	Substantial	57.76	Very high	12.5	672	4,083	4.01	2
		6	76.18	Substantial	33.62	Very high	8	490	3,821	3.76	1
		7	66.93	Moderate	39.66	Very high	20.69	525	3,808	3.56	4
		8	33.35	Partial	2.35	Low	50	222	1,103	0.94	0
		9	43.1	Partial	19.84	Very high	35.71	338	1,693	1.47	0
		10	83.62	Substantial	62.93	Very high	92.86	398	3,704	3.99	2
		11	73.34	Substantial	21.32	Very high	8	832	4,229	4.03	3
		12	41.5	Partial	19.75	Very high	28	635	4,010	3.33	3
C. maculatus	6	1	98.28	Near	3.45	Low	0	472	2,911	3.57	1

		2	96.55	Near	1.72	Low	0	290	3,308	3.44	1
		3	64.58	Moderate	32.05	Very high	30.77	1,300	6,793	6.04	4
		4	98.28	Near	0	Low	0	26	2,147	2.54	1
		5	64.5	Moderate	26.03	Very high	9.09	766	3,583	3.12	1
		6	92.63	Near	6.38	Medium	20	176	2,383	2.4	0
C. minutus	8	1	96.55	Near	0	Low	0	352	2,530	2.37	0
		2	98.28	Near	3.45	Low	0	390	2,671	3.01	1
		3	8.97	Partial	0	Low	0	328	1,740	1.44	3
		4	66.64	Moderate	0.16	Low	0	344	2,329	2.1	0
		5	96.55	Near	0	Low	0	74	2,393	2.5	1
		6	61.15	Moderate	0	Low	0	436	2,528	2.28	1
		7	97.41	Near	0	Low	0	116	2,170	2.51	1
		8	78.03	Substantial	40.99	Very high	9.26	1,072	5,110	4.38	2
C_pallens	10	1	94.83	Near	0	Low	0	253	1,840	1.97	0
		2	91.38	Near	0	Low	0	533	2,760	2.94	2
		3	96.55	Near	3.45	Low	0	162	2,736	2.79	1
		4	70.14	Substantial	20.69	Very high	22.22	993	4,940	4.99	5
		5	89.03	Substantial	53.94	Very high	23.73	1,300	6,840	6.16	8
		6	88.87	Substantial	28.45	Very high	4.76	472	3,815	3.76	3
		7	60.27	Moderate	10.61	High	36.36	451	2,463	2.12	2
		8	85.11	Substantial	0	Low	0	281	2,444	2.33	1
		9	69.09	Moderate	7.42	Medium	16.67	533	2,918	2.63	0
		10	83.62	Substantial	0.16	Low	0	322	2,522	2.84	0
C. pellans	7	1	100	Near	0	Low	0	403	2,303	2.37	1
		2	62.63	Moderate	20.73	Very high	5	918	2,904	3.1	5
		3	98.28	Near	0	Low	0	131	2,172	2.6	2
		4	97.41	Near	0	Low	0	36	4,504	4.12	1
		5	53.92	Moderate	16.09	Very high	11.54	825	2,801	2.58	2
		6	56.97	Moderate	25.86	Very high	33.33	845	4,280	3.92	1
		7	65.52	Moderate	3.45	Low	0	454	4,452	4.02	2
C. persimilis	6	1	98.28	Near	0.31	Low	0	475	5,164	5.07	1
		2	100	Near	13.95	High	0	623	3,314	3.54	7
		3	54.31	Moderate	18.55	Very high	7.14	722	2,148	2.52	0
		4	95.69	Near	0	Low	0	22	4,195	3.49	1
		5	69.76	Moderate	35.85	Very high	46.88	1,261	3,784	3.03	2
		6	78.68	Substantial	28.76	Very high	24.14	810	6,564	5.66	2
C. persimplex	11	1	98.28	Near	0	Low	0	121	1,836	1.94	0
		2	80.53	Substantial	24.87	Very high	0	1,052	3,906	4.1	0
		3	92.16	Near	5.17	Medium	0	1,293	4,905	5.9	0
		4	91.95	Near	0	Low	0	189	1,467	1.33	0
		5	100	Near	0	Low	0	192	2,020	2.11	0
		6	91.38	Near	0	Low	0	207	2,856	2.81	3
		7	100	Near	19.12	Very high	0	497	4,608	4.34	4
		8	81.03	Substantial	5.8	Medium	14.29	437	2,707	2.75	1
		9	81.35	Substantial	32.51	Very high	55.81	825	4,921	4.78	2
		10	54.39	Moderate	10.42	High	0	556	2,557	2.28	0
		11	84.48	Substantial	26.18	Very high	3.85	605	3,858	3.56	4
C. pusillus	5	1	54.94	Moderate	0	Low	0	281	3,727	3.59	0
		2	98.28	Near	6.9	Medium	0	429	2,381	2.73	4

		3	95.69	Near	0	Low	0	165	3,194	2.82	1
		4	78.45	Substantial	18.04	Very high	0	635	3,009	2.54	0
		5	62.54	Moderate	13.09	High	0	635	1,228	1.15	1
C. rohweri	22	1	98.28	Near	0	Low	0	29	1,583	1.64	0
		2	38.76	Partial	38.76	Very high	0	921	6,274	8.68	0
		3	96.55	Near	0	Low	0	47	1,729	1.82	0
		4	67.24	Moderate	12.07	High	4.35	3,378	18,614	31.63	2
		5	98.28	Near	10.97	High	51.06	147	3,052	3.22	6
		6	93.1	Near	0	Low	0	59	2,164	2.42	1
		7	87.3	Substantial	2.3	Low	50	108	2,148	2.24	1
		8	87.62	Substantial	76.65	Very high	29.17	313	4,518	4.46	1
		9	79.31	Substantial	53.45	Very high	20.45	424	5,174	5.36	4
		10	98.28	Near	0	Low	0	77	2,295	2.36	3
		11	100	Near	0	Low	0	31	2,004	2.15	1
		12	85.06	Substantial	22.88	Very high	39.13	202	3,421	3.46	3
		13	93.1	Near	3.45	Low	50	88	2,033	2.11	1
		14	63.79	Moderate	17.82	Very high	91.89	121	2,045	2.11	2
		15	69.83	Moderate	21.47	Very high	68.42	131	2,775	2.87	4
		16	88.09	Substantial	0	Low	0	90	2,073	2.16	1
		17	62.1	Moderate	23.98	Very high	4.65	436	2,152	2	0
		18	44.39	Partial	26.8	Very high	48.72	323	1,693	1.48	0
		19	62.93	Moderate	13.79	High	30	671	3,489	3.16	0
		20	22.42	Partial	8.31	Medium	0	14,539	46,227	71.17	1
		21	36.96	Partial	1.32	Low	0	620	2,492	1.92	0
		22	41.75	Partial	5.34	Medium	0	13,054	31,045	45.58	0
C. similimus	6	1	94.83	Near	5.17	Medium	25	328	2,123	1.99	0
		2	100	Near	8.78	Medium	0	946	4,107	4.89	0
		3	66.93	Moderate	0	Low	0	371	1,964	1.67	0
		4	85.19	Substantial	3.45	Low	100	275	2,249	2.34	0
		5	60.27	Moderate	21	Very high	12.5	803	4,703	4.1	3
		6	75.39	Substantial	24.5	Very high	7.32	826	4,667	3.95	4
C. spinosus	8	1	95.69	Near	1.72	Low	100	50	2,077	2.53	0
		2	87.93	Substantial	8.97	Medium	0	450	3,359	3.05	4
		3	77.89	Substantial	18.97	Very high	75	267	2,519	2.31	1
		4	41.38	Partial	5.17	Medium	33.33	173	2,059	1.9	1
		5	98.28	Near	0	Low	0	81	2,658	2.72	3
		6	54.2	Moderate	18.97	Very high	33.33	1,067	5,996	5.08	6
		7	100	Near	0	Low	0	514	4,141	4.46	3
		8	93.18	Near	0	Low	0	825	3,903	4.28	2
C. umbraculatus	14	1	3.45	Partial	0	Low	0	1,298	2,744	3.83	0
		2	63.79	Moderate	30.38	Very high	3.12	664	3,383	3.26	2
		3	93.03	Near	3.45	Low	33.33	332	4,114	3.83	4
		4	100	Near	0.86	Low	0	147	2,493	2.45	2
		5	98.28	Near	21	Very high	0	4,233	11,471	18.38	1
		6	98.28	Near	7.21	Medium	0	3,922	11,620	16.85	0
		7	88.79	Substantial	8.89	Medium	0	2,918	8,842	11.08	0
		8	79.31	Substantial	14.55	High	0	669	3,307	4.09	2
		9	94.83	Near	2.35	Low	0	753	4,373	5.11	2
		10	98.28	Near	7.47	Medium	18.75	158	2,106	2.13	0

		11	84.48	Substantial	0	Low	0	112	1,455	1.26	0
		12	76.49	Substantial	50	Very high	15.38	374	2,580	2.58	1
		13	100	Near	3.45	Low	50	109	1,957	2.27	0
		14	30.39	Partial	0.73	Low	0	5,345	12,816	17.88	0
C. varians PL005W	11	1	98.28	Near	2.38	Low	0	1,020	4,203	4.6	1
		2	93.1	Near	19.44	Very high	86.89	211	2,449	2.52	0
		3	85.58	Substantial	57.76	Very high	45.24	459	3,650	3.6	1
		4	98.28	Near	1.72	Low	100	253	2,957	2.9	4
		5	89.66	Substantial	0	Low	0	67	1,793	1.84	1
		6	88.97	Substantial	48.28	Very high	52.5	219	3,237	3.42	2
		7	75.08	Substantial	41.69	Very high	58.82	224	2,656	2.74	1
		8	98.28	Near	26.8	Very high	3.85	120	2,542	2.65	1
		9	79.31	Substantial	38.54	Very high	42.61	422	3,599	3.55	3
		10	87.93	Substantial	34.48	Very high	6.9	583	3,581	3.55	2
		11	46.08	Partial	31.03	Very high	64	480	3,651	3.53	2
C. varians PL010W	11	1	86.21	Substantial	0.34	Low	0	142	1,237	1.65	0
		2	53.29	Moderate	0	Low	0	136	1,842	1.7	0
		3	50	Moderate	0	Low	0	92	1,438	2.01	0
		4	42.95	Partial	0	Low	0	62	1,983	2.01	0
		5	97.41	Near	0	Low	0	45	2,475	2.04	0
		6	100	Near	1.72	Low	0	126	3,585	2.91	0
		7	98.28	Near	0	Low	0	112	1,586	2.3	2
		8	96.55	Near	0	Low	0	101	2,542	2.16	0
		9	94.83	Near	0	Low	0	84	2,079	1.99	1
		10	94.83	Near	0	Low	0	87	1,972	1.85	0
		11	87.93	Substantial	0	Low	0	63	1,845	1.49	1

The metagenomic bins were created by uploading each metagenome sequences into the Anvi'o version 5.5. This table contains for each *Cephalotes* metagenome the number of bins created, and for each bin it shows the percentage of completeness, the percentage of contamination, the percentage of strain heterogeneity, the number of contigs, the number of genes, and the number of BGCs found. The percentage of completeness, contamination and strain heterogeneity were obtained through the CheckM software. The number of contigs and the number of genes were obtained through the Anvi'o software. The number of BGC was obtained through the AntiSMASH software

Supplementary Table 4. BGCs types identified in the bacterial genomes.

Host	Symbiont	Symbiont BGC number	Type of cluster	Number of genes	Size of cluster (nt)
C. varians	Burkholderiales sp. Cv33a	Burkholderiales_Cv33a_1			
C. varians	Burkholderiales sp. Cv36	Burkholderiales_Cv36_1	Terpene	20	21,712
C. varians	Burkholderiales sp. Cv36	Burkholderiales_Cv36_2	Arylpoyene	27	25,774
C. varians	Burkholderiales sp. Cv44	Burkholderiales_Cv44_1	T1PKS	42	47,950
C. varians	Burkholderiales sp. Cv44	Burkholderiales_Cv44_2	NRP	27	33,193
C. varians	Burkholderiales sp. Cv44	Burkholderiales_Cv44_3	Betalactone	16	15,437
C. varians	Burkholderiales sp. Cv52	Burkholderiales_Cv52_1	Terpene	22	21,745
C. varians	Burkholderiales sp. Cv52	Burkholderiales_Cv52_2	Resorcinol	31	41,911
C. varians	Burkholderiales sp. Cv52	Burkholderiales_Cv52_3	T1PKS	28	34,252
C. varians	Burkholderiales sp. Cv52	Burkholderiales_Cv52_4	Ectoine	8	9,669
C. varians	*Cephaloticoccus canophilus* Cv41	*Cephaloticoccus_canophilus*_Cv41_1			
C. varians	Rhizobiales sp. JR021-5	Rhizobiales JR021-5_1			
C. varians	*Ventosimonas gracilis* Cv58	*Ventosimonas_gracilis*_Cv58_1	Arylpoyene	37	43,580
C. rohweri	Burkholderiales sp. Cag20	Burkholderiales_Cag20_1	T1PKS	36	36,938
C. rohweri	Burkholderiales sp. Cag20	Burkholderiales_Cag20_2	NRP	27	30,210
C. rohweri	Burkholderiales sp. Cag20	Burkholderiales_Cag20_3	Betalactone	20	22,669
C. rohweri	Burkholderiales sp. Cag20	Burkholderiales_Cag20_4	NRP	7	9,722
C. rohweri	Burkholderiales sp. Cag20	Burkholderiales_Cag20_5	NRP	34	39,296
C. rohweri	Burkholderiales sp. Cag25	Burkholderiales_Cag25_1	Betalactone	20	28,119
C. rohweri	Burkholderiales sp. Cag25	Burkholderiales_Cag25_2	T1PKS	34	47,910
C. rohweri	Burkholderiales sp. Cag25	Burkholderiales_Cag25_3	NRP	41	45,968
C. rohweri	Burkholderiales sp. Cag25	Burkholderiales_Cag25_4	NRP	32	27,559
C. rohweri	*Cephaloticoccus primus* Cag34	*Cephaloticoccus_primus*_Cag34_1	Arylpoyene	47	42,932
C. varians	*Ventosimonas* sp. Cag26	*Ventosimonas*_Cag26_1	Arylpoyene	31	26,327
C. rohweri	*Ventosimonas* sp. Cag26	*Ventosimonas*_Cag26_2	NRP	33	39,361
C. varians	*Ventosimonas* sp. Cag27	*Ventosimonas*_Cag27_1	NRP	34	36,361
C. rohweri	*Ventosimonas* sp. Cag27	*Ventosimonas*_Cag27_2	Arylpoyene	40	26,376
C. rohweri	*Ventosimonas* sp. Cag320	*Ventosimonas*_Cag320_1	NRP	18	28,447
C. rohweri	*Ventosimonas* sp. Cag320	*Ventosimonas*_Cag320_2	NRP	28	24,149
C. rohweri	Xanthomonadales sp. Cag60	Xanthomonadales_Cag60_1	NRP	53	44,406
C. rohweri	Xanthomonadales sp. Cag60	Xanthomonadales_Cag60_2	Ladderane	28	29,577
C. rohweri	Xanthomonadales sp. Cag60	Xanthomonadales_Cag60_3	NRP	45	53,610
C. varians	Xanthomonadales sp. Cag60	Xanthomonadales_Cag60_4	Siderophore	9	9,390
C. varians	Xanthomonadales sp. Cag60	Xanthomonadales_Cag60_5	Arylpoyene	43	38,815

This table contains the number of BGCs detected in each bacteria isolated from *C. varians* and *C. rohweri*, as well as the length and number of genes composing these BGCs. The colors of the cell represent the type of BGCs according to AntiSMASH color code

Supplementary Table 5. BGCs types identified in the metagenomic bins.

The colors of the cell represent the type of BGCs according to AntiSMASH color code.

**Geography**	**Host species**	**Bins**	**BGC ID in bin**	**Completeness bin (%)**	**CheckM completness class**	**Contamination bin (%)**	**CheckM contamination class**	**Strain heterogeneity bin (%)**	**Number contigs bin**	**Number genes bin**	**Type gene cluster in bin**	**Length cluster (nt)**	**Number of genes in cluster**	**Symbiont order**	**Symbiont family**	**Symbiont genus**
Peru	*C. angustus*	Bin 2	Bin 2	95.69	Near	0.00	Low	0.00	316	1 506.00	Arylpolyene	42 392.00	39	Burkholderiales	Enterobacteriaceae	Serratia
Brazil	*C. atratus*	Bin 9	Bin 9_2	81.43	Substantial	25.49	Very high	65.00	3 733.00	455	Arylpolyene	23 687.00	31	Burkholderiales	Burkholderiaceae	Ralstonia
Brazil	*C. atratus*	Bin 2	Bin 2	96.55	Near	3.76	Low	0.00	2 760.00	8 783.00	LAP	6 492.00	9	Bacillales	Listeriaceae	Listeria
Brazil	*C. atratus*	Bin 11	Bin 11_4	80.06	Substantial	32.37	Very high	38.46	4 765.00	798	Arylpolyene	6 235.00	6	Burkholderiales	Comamonadaceae	Verminephrobacter
Brazil	*C. atratus*	Bin 12	Bin 12	60.34	Moderate	21.22	Very high	18.00	2 575.00	523	Arylpolyene	24 572.00	35	Burkholderiales	Alcaligenaceae	Achromobacter
Brazil	*C. atratus*	Bin 1	Bin 1	98.28	Near	2.77	Low	0.00	8 646.00	3 112.00	Bacteriocin	8 308.00	15	Acholeplasmatales	Acholeplasmataceae	Acholeplasma
Brazil	*C. atratus*	Bin 10	Bin 10_2	68.97	Moderate	31.56	Very high	4.44	4 862.00	910	Hserlactone	6 118.00	8	Pseudomonadales	Pseudomonadaceae	Pseudomonas
Brazil	*C. atratus*	Bin 11	Bin 11_6	80.06	Substantial	32.37	Very high	38.46	4 765.00	798	Arylpolyene-Ladderane	6 994.00	8	Enterobacteriales	Enterobacteriaceae	Proteus
Brazil	*C. atratus*	Bin 10	Bin 10_1	68.97	Moderate	31.56	Very high	4.44	4 862.00	910	NRP	6 919.00	7	Burkholderiales	Burkholderiaceae	Burkholderia
Brazil	*C. atratus*	Bin 7	Bin 7	81.58	Substantial	2.07	Low	0.00	2 115.00	275	Terpene	12 499.00	12	Burkholderiales	Alcaligenaceae	Bordetella
Brazil	*C. atratus*	Bin 9	Bin 9_1	81.43	Substantial	25.49	Very high	65.00	3 733.00	455	Terpene	12 633.00	12	Burkholderiales	Comamonadaceae	Acidovorax
Brazil	*C. clypeatus*	Bin 2	Bin 2_3	94.83	Near	31.90	Very high	0.00	632	5 449.00	Arylpolyene	32 535.00	35	Burkholderiales	Comamonadaceae	Melaminivora
Brazil	*C. clypeatus*	Bin 2	Bin 2_4	94.83	Near	31.90	Very high	0.00	632	5 449.00	LAP	9 615.00	10	Xanthomonadales	Xanthomonadaceae	Lysobacter
Brazil	*C. clypeatus*	Bin 2	Bin 2_1	94.83	Near	31.90	Very high	0.00	632	5 449.00	Betalactone	25 272.00	28	Burkholderiales	Burkholderiaceae	Cupriavidus
Brazil	*C. clypeatus*	Bin 3	Bin 3_1	99.50	Near	49.95	Very high	31.58	717	4 724.00	Ladderane	19 231.00	20	Enterobacteriales	Enterobacteriaceae	Serratia
Brazil	*C. clypeatus*	Bin 5	Bin 5_2	80.41	Substantial	1.72	Low	0.00	508	2 165.00	Lanthipeptide	6 072.00	4	Xanthomonadales	Xanthomonadaceae	Lysobacter
Brazil	*C. clypeatus*	Bin 3	Bin 3_2	99.50	Near	49.95	Very high	31.58	717	4 724.00	NRP	8 602.00	8	Xanthomonadales	Xanthomonadaceae	Stenotrophomonas
Brazil	*C. clypeatus*	Bin 5	Bin 5_1	80.41	Substantial	1.72	Low	0.00	508	2 165.00	Siderophore	5 204.00	3	Rhizobiales	Rhizobiaceae	Sinorhizobium
Brazil	*C. clypeatus*	Bin 2	Bin 2_2	94.83	Near	31.90	Very high	0.00	632	5 449.00	Terpene	18 172.00	23	Enterobacteriales	Enterobacteriaceae	Erwinia
Brazil	*C. eduarduli*	Bin 8	Bin 8_1	14.58	Partial	1.72	Low	100.00	736	4 462.00	Acyl amino acids	11 694.00	11	Rhizobiales	Methylobacteriaceae	Methylobacterium
Brazil	*C. eduarduli*	Bin 3	Bin 3	98.28	Near	6.90	Medium	0.00	181	3 023.00	Arylpolyene	6 560.00	6	Burkholderiales	Alcaligenaceae	Achromobacter
Brazil	*C. eduarduli*	Bin 6	Bin 6_2	79.00	Substantial	27.90	Very high	52.63	588	4 057.00	Arylpolyene	5 347.00	9	Pseudomonadales	Pseudomonadaceae	Pseudomonas
Brazil	*C. eduarduli*	Bin 8	Bin 8_3	14.58	Partial	1.72	Low	100.00	736	4 462.00	Butyrolactone	7 902.00	10	Burkholderiales	Alcaligenaceae	Alcaligenes
Brazil	*C. eduarduli*	Bin 8	Bin 8_5	14.58	Partial	1.72	Low	100.00	736	4 462.00	Butyrolactone	5 545.00	6	Burkholderiales	Alcaligenaceae	Alcaligenes

Brazil	*C. eduarduli*	Bin 1	Bin 1	96.55	Near	0.00	Low	0.00	448	2 497.00	Furan	14 566.00	17	Flavobacteriales	Flavobacteriaceae	Elizabethkingia
Brazil	*C. eduarduli*	Bin 6	Bin 6_1	79.00	Substantial	27.90	Very high	52.63	588	4 057.00	Ladderane	15 012.00	13	Enterobacteriales	Enterobacteriaceae	Serratia
Brazil	*C. eduarduli*	Bin 8	Bin 8_2	14.58	Partial	1.72	Low	100.00	736	4 462.00	NRP	16 384.00	6	Enterobacteriales	Yersiniaceae	Yersinia
Brazil	*C. eduarduli*	Bin 4	Bin 4_2	86.41	Substantial	14.81	High	54.55	640	4 304.00	NRP	21 928.00	21	Pseudomonadales	Pseudomonadaceae	Pseudomonas
Brazil	*C. eduarduli*	Bin 8	Bin 8_4	14.58	Partial	1.72	Low	100.00	736	4 462.00	NRP	5 624.00	6	Pseudomonadales	Pseudomonadaceae	Pseudomonas
Brazil	*C. eduarduli*	Bin 6	Bin 6_3	79.00	Substantial	27.90	Very high	52.63	588	4 057.00	Resorcinol	20 058.00	33	Xanthomonadales	Xanthomonadaceae	Stenotrophomonas
Brazil	*C. eduarduli*	Bin 8	Bin 8_6	14.58	Partial	1.72	Low	100.00	736	4 462.00	Siderophore	8 042.00	7	Burkholderiales	Alcaligenaceae	Achromobacter
Brazil	*C. eduarduli*	Bin 4	Bin 4_1	86.41	Substantial	14.81	High	54.55	640	4 304.00	T1PK	15 929.00	15	Burkholderiales	Alcaligenaceae	Achromobacter
Brazil	*C. eduarduli*	Bin 4	Bin 4_4	86.41	Substantial	14.81	High	54.55	640	4 304.00	Terpene	10 275.00	14	Rhizobiales	Brucellaceae	Ochrobactrum
Brazil	*C. grandinosus*	Bin 4	Bin 4_2	82.45	Substantial	24.48	Very high	10.53	425	1 968.00	Arylpolyene	6 092.00	8	Burkholderiales	Alcaligenaceae	Achromobacter
Brazil	*C. grandinosus*	Bin 10	Bin 10_1	83.62	Substantial	62.93	Very high	92.86	231	2 414.00	Arylpolyene	19 882.00	20	Burkholderiales	Alcaligenaceae	Achromobacter
Brazil	*C. grandinosus*	Bin 10	Bin 10_2	83.62	Substantial	62.93	Very high	92.86	231	2 414.00	Arylpolyene	23 001.00	31	Burkholderiales	Alcaligenaceae	Achromobacter
Brazil	*C. grandinosus*	Bin 4	Bin 4_4	82.45	Substantial	24.48	Very high	10.53	425	1 968.00	Arylpolyene	10 418.00	8	Pseudomonadales	Pseudomonadaceae	Pseudomonas
Brazil	*C. grandinosus*	Bin 11	Bin 11_1	73.34	Substantial	62.93	Very high	92.86	414	2 158.00	Arylpolyene	9 981.00	10	Pseudomonadales	Pseudomonadaceae	Pseudomonas
Brazil	*C. grandinosus*	Bin 11	Bin 11_3	73.34	Substantial	62.93	Very high	92.86	414	2 158.00	Arylpolyene	6 212.00	9	Pseudomonadales	Pseudomonadaceae	Pseudomonas
Brazil	*C. grandinosus*	Bin 11	Bin 11_2	73.34	Substantial	62.93	Very high	92.86	173	778	Bacteriocin	6 103.00	6	Xanthomonadales	Xanthomonadaceae	Xanthomonas
Brazil	*C. grandinosus*	Bin 6	Bin 6_1	76.18	Substantial	33.62	Very high	8.00	234	2 272.00	Betalactone	21 831.00	23	Burkholderiales	Alcaligenaceae	Bordetella
Brazil	*C. grandinosus*	Bin 7	Bin 7_3	66.93	Moderate	39.66	Very high	20.69	277	2 410.00	Betalactone	13 647.00	15	Burkholderiales	Alcaligenaceae	Achromobacter
Brazil	*C. grandinosus*	Bin 4	Bin 4_6	82.45	Substantial	24.48	Very high	10.53	425	1 968.00	NRP	15 960.00	11	Bacillales	Paenibacillaceae	Paenibacillus
Brazil	*C. grandinosus*	Bin 7	Bin 7_4	66.93	Moderate	39.66	Very high	20.69	277	2 410.00	NRP	9 572.00	8	Burkholderiales	Alcaligenaceae	Alcaligenes
Brazil	*C. grandinosus*	Bin 12	Bin 12_4	41.50	Partial	19.75	Very high	28.00	311	1 926.00	NRP	7 491.00	3	Burkholderiales	Burkholderiaceae	Burkholderia
Brazil	*C. grandinosus*	Bin 4	Bin 4_3	82.45	Substantial	24.48	Very high	10.53	425	1 968.00	NRP	19 174.00	7	Pseudomonadales	Pseudomonadaceae	Pseudomonas
Brazil	*C. grandinosus*	Bin 4	Bin 4_9	82.45	Substantial	24.48	Very high	10.53	425	1 968.00	NRP	7 884.00	4	Pseudomonadales	Pseudomonadaceae	Pseudomonas
Brazil	*C. grandinosus*	Bin 12	Bin 12_1	41.50	Partial	19.75	Very high	28.00	311	1 926.00	NRP	11 820.00	11	Rhizobiales	Brucellaceae	Brucella
Brazil	*C. grandinosus*	Bin 4	Bin 4_1	82.45	Substantial	24.48	Very high	10.53	425	1 968.00	Phenazine	5 441.00	5	Pseudomonadales	Pseudomonadaceae	Pseudomonas
Brazil	*C. grandinosus*	Bin 7	Bin 7_2	66.93	Moderate	39.66	Very high	20.69	248	1 398.00	Resorcinol	11 807.00	11	Burkholderiales	Alcaligenaceae	Achromobacter
Brazil	*C. grandinosus*	Bin 3	Bin 3	96.55	Near	0.00	Low	0.00	90	2 357.00	Resorcinol	32 027.00	33	Xanthomonadales	Xanthomonadaceae	Xanthomonas
Brazil	*C. grandinosus*	Bin 5	Bin 5_1	88.79	Substantial	57.76	Very high	12.50	267	2 015.00	T1PK	9 635.00	7	Burkholderiales	Alcaligenaceae	Bordetella
Brazil	*C. grandinosus*	Bin 12	Bin 12_2	41.50	Partial	19.75	Very high	28.00	324	2 084.00	T1PK	30 486.00	26	Burkholderiales	Alcaligenaceae	Bordetella
Brazil	*C. grandinosus*	Bin 5	Bin 5_5	88.79	Substantial	57.76	Very high	12.50	267	2 015.00	Terpene	12 750.00	16	Burkholderiales	Comamonadaceae	Comamonas

Brazil	*C. grandinosus*	Bin 7	Bin 7_5	66.93	Moderate	39.66	Very high	20.69	248	1 398.00	Terpene	7 469.00	8	Burkholderiales	Alcaligenaceae	Bordetella
Brazil	*C. grandinosus*	Bin 4	Bin 4_12	82.45	Substantial	24.48	Very high	10.53	425	1 968.00	LAP	9 681.00	12	Xanthomonadales	Xanthomonadaceae	Xanthomonas
Brazil	*C. grandinosus*	Bin 4	Bin 4_10	82.45	Substantial	24.48	Very high	10.53	425	1 968.00	Terpene	6 123.00	5	Pseudomonadales	Moraxellaceae	Acinetobacter
Brazil	*C. maculatus*	Bin 4	Bin 4	98.28	Near	0.00	Low	0.00	26	2 147.00	Arylpolyene	42 670.00	38	Verrucomicrobiales	Opitutaceae	Opitutus
Brazil	*C. maculatus*	Bin 3	Bin 3_6	64.58	Moderate	32.05	Very high	30.77	1 300.00	6 793.00	Arylpolyene	5 021.00	6	Xanthomonadales	Xanthomonadaceae	Xanthomonas
Brazil	*C. maculatus*	Bin 3	Bin 3_5	64.58	Moderate	32.05	Very high	30.77	1 300.00	6 793.00	NRP	18 724.00	7	Bacillales	Paenibacillaceae	Brevibacillus
Brazil	*C. maculatus*	Bin 5	Bin 5_3	64.50	Moderate	26.03	Very high	9.09	766	3 583.00	NRP	6 888.00	2	Pseudomonadales	Pseudomonadaceae	Pseudomonas
Brazil	*C. maculatus*	Bin 3	Bin 3_1	64.58	Moderate	32.05	Very high	30.77	1 300.00	6 793.00	NRP-T1PK	21 264.00	11	Burkholderiales	Burkholderiaceae	Ralstonia/Burkholderia
Brazil	*C. maculatus*	Bin 2	Bin 2	96.55	Near	1.72	Low	0.00	290	3 308.00	Resorcinol	32 905.00	36	Burkholderiales	Alcaligenaceae	Alacaligenes
Brazil	*C. maculatus*	Bin 3	Bin 3_2	64.58	Moderate	32.05	Very high	30.77	1 300.00	6 793.00	LAP	14 213.00	13	Xanthomonadales	Xanthomonadaceae	Stenotrophomonas
Brazil	*C. maculatus*	Bin 1	Bin 1	98.28	Near	3.45	Low	0.00	472	2 911.00	Terpene	20 923.00	17	Rhizobiales	Bartonellaceae	Bartonella
Brazil	*C. minutus*	Bin 6	Bin 6_1	61.15	Moderate	0.00	Low	0.00	436	2 528.00	Arylpolyene	14 647.00	15	Burkholderiales	Alcaligenaceae	Achromobacter
Brazil	*C. minutus*	Bin 7	Bin 7	97.41	Near	0.00	Low	0.00	116	2 170.00	Arylpolyene	25 728.00	39	Burkholderiales	Alcaligenaceae	Achromobacter
Brazil	*C. minutus*	Bin 5	Bin 5	96.55	Near	0.00	Low	0.00	74	2 393.00	Arylpolyene	43 072.00	57	Xanthomonadales	Xanthomonadaceae	Stenotropomonas
Brazil	*C. minutus*	Bin 8	Bin 8_4	78.03	Substantial	40.99	Very high	9.26	1 072.00	5 110.00	Butyrolactone	9 664.00	13	Pseudomonadales	Pseudomonadaceae	Pseudomonas
Brazil	*C. minutus*	Bin 3	Bin 3_2	8.97	Partial	0.00	Low	0.00	328	1 740.00	Lanthipeptide	5 678.00	5	Xanthomonadales	Xanthomonadaceae	Stenotropomonas
Brazil	*C. minutus*	Bin 3	Bin 3_3	8.97	Partial	0.00	Low	0.00	328	1 740.00	LAP	8 413.00	9	Enterobacteriales	Enterobacteriaceae	Serratia
Brazil	*C. minutus*	Bin 2	Bin 2	98.28	Near	3.45	Low	0.00	390	2 671.00	NRP	7 176.00	5	Desulfovibrionales	Desulfovibrionaceae	Desulfovibrio
Brazil	*C. minutus*	Bin 8	Bin 8_2	78.03	Substantial	40.99	Very high	9.26	1 072.00	5 110.00	NRP	7 334.00	6	Pseudomonadales	Pseudomonadaceae	Pseudomonas
Brazil	*C. minutus*	Bin 3	Bin 3_1	8.97	Partial	0.00	Low	0.00	328	1 740.00	NRP	9 249.00	8	Rhizobiales	Brucellaceae	Brucella
Brazil	*C. pallens*	Bin 5	Bin 5_10	89.03	Substantial	53.94	Very high	23.73	1 300.00	6 840.00	Arylpolyene	7 002.00	8	Burkholderiales	Comamonadaceae	Verminephrobacter
Brazil	*C. pallens*	Bin 5	Bin 5_11	89.03	Substantial	53.94	Very high	23.73	1 300.00	6 840.00	Arylpolyene	5 011.00	4	Burkholderiales	Comamonadaceae	Verminephrobacter
Brazil	*C. pallens*	Bin 2	Bin 2_2	91.38	Near	0.00	Low	0.00	533	2 760.00	Arylpolyene	9 255.00	13	Campylobacterales	Campylobacteraceae	Arcobacter
Brazil	*C. pallens*	Bin 6	Bin 6_4	88.87	Substantial	28.45	Very high	4.76	472	3 815.00	Ectoine	7 791.00	7	Rhizobiales	Brucellaceae	Ochrobactrum
Brazil	*C. pallens*	Bin 4	Bin 4_1	70.14	Substantial	20.69	Very high	22.22	993	4 940.00	Lanthipeptide	19 732.00	25	Sphingobacteriales	Sphingobacteriaceae	Sphingobacterium
Brazil	*C. pallens*	Bin 5	Bin 5_2	89.03	Substantial	53.94	Very high	23.73	1 300.00	6 840.00	NRP	21 156.00	17	Burkholderiales	Burkholderiaceae	Burkholderia
Brazil	*C. pallens*	Bin 5	Bin 5_7	89.03	Substantial	53.94	Very high	23.73	1 300.00	6 840.00	NRP	14 425.00	6	Burkholderiales	Alacaligenaceae	Achromobacter
Brazil	*C. pallens*	Bin 7	Bin 7_1	60.27	Moderate	10.61	High	36.36	451	2 463.00	NRP	6 271.00	7	Burkholderiales	Alacaligenaceae	Alcaligenes
Brazil	*C. pallens*	Bin 5	Bin 5_5	89.03	Substantial	53.94	Very high	23.73	1 300.00	6 840.00	NRP	8 496.00	3	Pseudomonadales	Pseudomonadaceae	Pseudomonas
Brazil	*C. pallens*	Bin 5	Bin 5_3	89.03	Substantial	53.94	Very high	23.73	1 300.00	6 840.00	NRP	19 586.00	17	Rhizobiales	Brucellaceae	Brucella

Brazil	*C. pallens*	Bin 4	Bin 4_4	70.14	Substantial	20.69	Very high	22.22	993	4 940.00	Phenazine	11 250.00	11	Pseudomonadales	Pseudomonadaceae	Pseudomonas
Brazil	*C. pallens*	Bin 4	Bin 4_5	70.14	Substantial	20.69	Very high	22.22	993	4 940.00	Phenazine	5 071.00	7	Pseudomonadales	Pseudomonadaceae	Pseudomonas
Brazil	*C. pallens*	Bin 5	Bin 5_6	89.03	Substantial	53.94	Very high	23.73	1 300.00	6 840.00	Resorcinol	10 397.00	9	Burkholderiales	Alacaligenaceae	Achromobacter
Brazil	*C. pallens*	Bin 2	Bin 2_1	91.38	Near	0.00	Low	0.00	533	2 760.00	Resorcinol	9 029.00	11	Campylobacterales	Campylobacteraceae	Arcobacter
Brazil	*C. pallens*	Bin 3	Bin 3	96.55	Near	3.45	Low	0.00	162	2 736.00	Resorcinol	28 165.00	28	Xanthomonadales	Xanthomonadaceae	Pseudoxanthomonas
Brazil	*C. pallens*	Bin 4	Bin 4_6	70.14	Substantial	20.69	Very high	22.22	993	4 940.00	Siderophore	8 875.00	9	Burkholderiales	Alacaligenaceae	Achromobacter
Brazil	*C. pallens*	Bin 5	Bin 5_4	89.03	Substantial	53.94	Very high	23.73	1 300.00	6 840.00	T1PK	9 854.00	2	Burkholderiales	Burkholderiaceae	Ralstonia
Brazil	*C. pallens*	Bin 7	Bin 7_2	60.27	Moderate	10.61	High	36.36	451	2 463.00	T1PK	6 178.00	2	Burkholderiales	Alacaligenaceae	Bordetella
Brazil	*C. pallens*	Bin 6	Bin 6_1	88.87	Substantial	23.45	Very high	4.76	472	3 815.00	Terpene	10 683.00	12	Burkholderiales	Alacaligenaceae	Bordetella
Brazil	*C. pallens*	Bin 6	Bin 6_2	88.87	Substantial	23.45	Very high	4.76	472	3 815.00	Terpene	7 225.00	9	Burkholderiales	Alacaligenaceae	Bordetella
Brazil	*C. pallens*	Bin 8	Bin 8	85.11	Substantial	0.00	Low	0.00	281	2 444.00	Terpene	13 242.00	13	Vibrionales	Vibrionaceae	Vibrio
Brazil	*C. pallens*	Bin 4	Bin 4_2	70.14	Substantial	20.69	Very high	22.22	993	4 940.00	Thiopeptide	6 791.00	7	Bacillales	Bacillaceae	Bacillus
Brazil	*C. pellans*	Bin 6	Bin 6_2	56.97	Moderate	25.86	Very high	33.33	845	4 452.00	Arylpolyene	6 881.00	9	Pseudomonadales	Pseudomonadaceae	Pseudomonas
Brazil	*C. pellans*	Bin 1	Bin 1	100.00	Near	0.00	Low	0.00	403	2 904.00	Arylpolyene-Resorcinol	45 905.00	49	Campylobacterales	Campylobacteraceae	Arcobacter
Brazil	*C. pellans*	Bin 2	Bin 2_5	62.63	Moderate	20.73	Very high	5.00	918	4 504.00	Butyrolactone	5 231.00	7	Actinomycetales	Streptomycetaceae	Streptomyces
Brazil	*C. pellans*	Bin 2	Bin 2_4	62.63	Moderate	20.73	Very high	5.00	918	4 504.00	Butyrolactone	5 550.00	8	Pseudomonadales	Pseudomonadaceae	Pseudomonas
Brazil	*C. pellans*	Bin 7	Bin 7_1	65.52	Moderate	3.45	Low	0.00	454	2 801.00	Ectoine	6 917.00	8	Rhizobiales	Brucellaceae	Ochrobactrum
Brazil	*C. pellans*	Bin 5	Bin 5_2	53.92	Moderate	16.09	Very high	11.54	825	4 280.00	NRP	12 865.00	4	Neisseriales	Neisseriaceae	Chromobacterium
Brazil	*C. pellans*	Bin 2	Bin 2_1	62.63	Moderate	20.73	Very high	5.00	918	4 504.00	NRP	5 644.00	4	Pseudomonadales	Moraxellaceae	Acinetobacter
Brazil	*C. pellans*	Bin 5	Bin 5_1	53.92	Moderate	16.09	Very high	11.54	825	4 280.00	NRP	13 491.00	12	Pseudomonadales	Pseudomonadaceae	Pseudomonas
Brazil	*C. pellans*	Bin 4	Bin 4	97.41	Near	0.00	Low	0.00	36	2 172.00	Phenazine	20 495.00	19	Pseudomonadales	Pseudomonadaceae	Pseudomonas
Brazil	*C. pellans*	Bin 3	Bin 3_1	98.28	Near	0.00	Low	0.00	131	2 303.00	Resorcinol	8 811.00	10	Xanthomonadales	Xanthomonadaceae	Stenotrophomonas
Brazil	*C. pellans*	Bin 3	Bin 3_2	98.28	Near	0.00	Low	0.00	131	2 303.00	Resorcinol	9 855.00	9	Xanthomonadales	Xanthomonadaceae	Stenotrophomonas
Brazil	*C. pellans*	Bin 2	Bin 2_7	62.63	Moderate	20.73	Very high	5.00	918	4 504.00	Siderophore	9 526.00	11	Burkholderiales	Alcaligenaceae	Achromobacter
Brazil	*C. pellans*	Bin 7	Bin 7_2	65.52	Moderate	3.45	Low	0.00	454	2 801.00	Terpene	6 474.00	8	Burkholderiales	Alcaligenaceae	Achromobacter
Brazil	*C. pellans*	Bin 2	Bin 2_3	62.63	Moderate	20.73	Very high	5.00	918	4 504.00	Thiopeptide	11 018.00	8	Bacillales	Bacillaceae	Bacillus
Brazil	*C. persimilis*	Bin 4	Bin 4	95.69	Near	0.00	Low	0.00	22	2.148	Arylpolyene	42 873.00	42	Opitutales	Opitutaceae	Cephaloticoccus
Brazil	*C. persimilis*	Bin 5	Bin 5_1	69.76	Moderate	35.85	Very high	46.88	1 261.00	6.564	Arylpolyene	9 360.00	12	Pseudomonadales	Pseudomonadaceae	Pseudomonas
Brazil	*C. persimilis*	Bin 1	Bin 1	98.28	Near	0.31	Low	0.00	475	3.314	Arylpolyene-Betalactone-Resorcinol	63 930.00	68	Campylobacterales	Campylobacteraceae	Arcobacter

Brazil	*C. persimilis*	Bin 6	Bin 6_2	76.68	Substantial	28.76	Very high	24.14	810	4.195	Ectoine	10 333.00	9	Burkholderiales	Alcaligenaceae	Bordetella
Brazil	*C. persimilis*	Bin 2	Bin 2_5	100.00	Near	13.95	High	0.00	623	5.164	Lanthipeptide	14 029.00	14	Flavobacteriales	Flavobacteriaceae	Kordia
Brazil	*C. persimilis*	Bin 5	Bin 5_3	69.76	Moderate	35.85	Very high	46.88	1 261.00	6.564	NRP	10 502.00	8	Burkholderiales	Alcaligenaceae	Achromobacter
Brazil	*C. persimilis*	Bin 2	Bin 2_1	100.00	Near	13.95	High	0.00	623	5.164	LAP	11 913.00	13	Xanthomonadales	Xanthomonadaceae	Xanthomonas
Brazil	*C. persimilis*	Bin 2	Bin 2_2	100.00	Near	13.95	High	0.00	623	5.164	NRP	14 699.00	13	Nostocales	Nostocaceae	Cylindrospermum
Brazil	*C. persimilis*	Bin 2	Bin 2_6	100.00	Near	13.95	High	0.00	623	5.164	NRP	11 940.00	5	Oscillatoriales	Microcoleaceae	Microcoleus
Brazil	*C. persimilis*	Bin 2	Bin 2_3	100.00	Near	13.95	High	0.00	623	5.164	NRP	10 617.00	4	Pseudomonadales	Pseudomonadaceae	Pseudomonas
Brazil	*C. persimilis*	Bin 2	Bin 2_4	100.00	Near	13.95	High	0.00	623	5.164	Resorcinol	22 365.00	21	Xanthomonadales	Xanthomonadaceae	Xanthomonas
Brazil	*C. persimilis*	Bin 2	Bin 2_8	100.00	Near	13.95	High	0.00	623	5.164	Siderophore	9 495.00	8	Burkholderiales	Alcaligenaceae	Achromobacter
Brazil	*C. persimilis*	Bin 6	Bin 6_1	78.68	Substantial	28.76	Very high	24.14	810	4.195	T1PK	6 869.00	7	Burkholderiales	Alcaligenaceae	Achromobacter
Peru	*C. persimplex*	Bin 6	Bin 6_1	91.38	Near	0.00	Low	0.00	207	2 856.00	Arylpolyene	25 746.00	42	Burkholderiales	Comamonadaceae	Alicycliphilus
Peru	*C. persimplex*	Bin 8	Bin 8_2	81.03	Substantial	5.80	Medium	14.29	437	2 707.00	Arylpolyene	5 178.00	6	Burkholderiales	Alcaligenaceae	Achromobacter
Peru	*C. persimplex*	Bin 11	Bin 11_4	84.48	Substantial	26.18	Very high	3.85	605	3.56	Arylpolyene	6 553.00	10	Burkholderiales	Alcaligenaceae	Achromobacter
Peru	*C. persimplex*	Bin 7	Bin 7_1	100.00	Near	19.12	Very high	0.00	497	4 608.00	Arylpolyene	35 413.00	36	Pseudomonadales	Pseudomonadaceae	Pseudomonas
Peru	*C. persimplex*	Bin 9	Bin 9_1	81.35	Substantial	32.51	Very high	55.81	825	4 921.00	Arylpolyene	22 142.00	29	Xanthomonadales	Xanthomonadaceae	Stenotrophomonas
Peru	*C. persimplex*	Bin 7	Bin 7_2	100.00	Near	19.12	Very high	0.00	497	4 608.00	Arylpolyene-Ladderane	45 003.00	40	Pseudomonadales	Pseudomonadaceae	Pseudomonas
Peru	*C. persimplex*	Bin 9	Bin 9_5	81.35	Substantial	32.51	Very high	55.81	825	4 921.00	Arylpolyene-Ladderane	5 725.00	4	Pseudomonadales	Pseudomonadaceae	Pseudomonas
Peru	*C. persimplex*	Bin 7	Bin 7_4	100.00	Near	19.12	Very high	0.00	497	4 608.00	Bacteriocin	7 647.00	7	Flavobacteriales	Flavobacteriaceae	Elizabethkingia
Peru	*C. persimplex*	Bin 7	Bin 7_5	100.00	Near	19.12	Very high	0.00	497	4 608.00	NRP	5 145.00	4	Desulfovibrionales	Desulfovibrionaceae	Desulfovibrio
Peru	*C. persimplex*	Bin 11	Bin 11_3	84.48	Substantial	26.18	Very high	3.85	605	3.56	NRP-T1PK	16 353.00	4	Enterobacteriales	Pectobacteriaceae	Brenneria
Peru	*C. persimplex*	Bin 11	Bin 11_1	84.48	Substantial	26.18	Very high	3.85	605	3.56	Resorcinol	8 229.00	9	Burkholderiales	Alcaligenaceae	Achromobacter
Peru	*C. persimplex*	Bin 6	Bin 6_2	91.38	Near	0.00	Low	0.00	207	2 856.00	Terpene	11 095.00	10	Burkholderiales	Comamonadaceae	Alicycliphilus
Peru	*C. persimplex*	Bin 6	Bin 6_3	91.38	Near	0.00	Low	0.00	207	2 856.00	Terpene	10 829.00	10	Burkholderiales	Comamonadaceae	Acidovorax
Peru	*C. persimplex*	Bin 11	Bin 11_2	84.48	Substantial	26.18	Very high	3.85	605	3.56	Terpene	17 690.00	19	Burkholderiales	Alcaligenaceae	Achromobacter
Brazil	*C. pusillus*	Bin 2	Bin 2_1	98.28	Near	6.90	Medium	0.00	429	3 727.00	Arylpolyene	20 582.00	25	Pseudomonadales	Pseudomonadaceae	Pseudomonas
Brazil	*C. pusillus*	Bin 3	Bin 3	95.69	Near	0.00	Low	0.00	165	2 381.00	Arylpolyene	24 174.00	23	Verrucomicrobiales	Opitutaceae	Lacunisphaera
Brazil	*C. pusillus*	Bin 2	Bin 2_2	98.28	Near	6.90	Medium	0.00	429	3 727.00	Butyrolactone	5 470.00	7	Rhodocyclales	Zoogloeaceae	Thauera
Brazil	*C. pusillus*	Bin 2	Bin 2_3	98.28	Near	6.90	Medium	0.00	429	3 727.00	Furan	5 003.00	9	Sphingobacteriales	Sphingobacteriaceae	Sphingobacterium
Brazil	*C. pusillus*	Bin 2	Bin 2_4	98.28	Near	6.90	Medium	0.00	429	3 727.00	Lanthipeptide	8 874.00	6	Sphingobacteriales	Sphingobacteriaceae	Pedobacter
Brazil	*C. pusillus*	Bin 5	Bin 5_1	62.54	Moderate	13.09	High	0.00	635	3 009.00	NRP	19 615.00	8	Neisseriales	Neisseriaceae	Chromobacterium

USA	*C. rohweri*	Bin 5	Bin 5_5	98.28	Near	10.97	High	51.06	3 052.00	147	Arylpolyene	8 431.00	8	Pseudomonadales	Ventosimonadaceae	Ventosimonas
USA	*C. rohweri*	Bin 8	Bin 8	87.62	Substantial	76.65	Very high	29.17	4 518.00	313	Arylpolyene	24 839.00	31	Pseudomonadales	Pseudomonadaceae	Pseudomonas
USA	*C. rohweri*	Bin 4	Bin 4_2	67.24	Moderate	12.07	High	4.35	18.614	3 378.00	Arylpolyene	6 739.00	7	Rhizobiales	Bartonellaceae	Bartonella
USA	*C. rohweri*	Bin 5	Bin 5_2	98.28	Near	10.97	High	51.06	3 052.00	147	Arylpolyene	29 483.00	27	Rhizobiales	Bartonellaceae	Bartonella
USA	*C. rohweri*	Bin 13	Bin 13	93.10	Near	3.45	Low	50.00	2 033.00	88	Betalactone	12 898.00	17	Burkholderiales	Alcaligenaceae	Achromobacter
USA	*C. rohweri*	Bin 15	Bin 15_3	69.83	Moderate	21.47	Very high	68.42	2 775.00	131	Betalactone	17 844.00	13	Burkholderiales	Alcaligenaceae	Achromobacter
USA	*C. rohweri*	Bin 12	Bin 12_3	85.06	Substantial	22.88	Very high	39.13	3 421.00	202	Betalactone	16 867.00	19	Pseudomonadales	Pseudomonadaceae	Pseudomonas
USA	*C. rohweri*	Bin 15	Bin 15_2	69.83	Moderate	21.47	Very high	68.42	2 775.00	131	Betalactone	22 283.00	21	Pseudomonadales	Pseudomonadaceae	Pseudomonas
USA	*C. rohweri*	Bin 9	Bin 9_2	79.31	Substantial	53.45	Very high	20.45	5 174.00	424	Ladderane	13 287.00	9	Xanthomonadales	Xanthomonadaceae	Xanthomonas
USA	*C. rohweri*	Bin 9	Bin 9_3	79.31	Substantial	53.45	Very high	20.45	5 174.00	424	Ladderane	18 057.00	18	Enterobacteriales	Enterobacteriaceae	Serratia
USA	*C. rohweri*	Bin 5	Bin 5_6	98.28	Near	10.97	High	51.06	3 052.00	147	Lanthipeptide	6 741.00	6	Xanthomonadales	Xanthomonadaceae	Stenotrophomonas
USA	*C. rohweri*	Bin 5	Bin 5_3	98.28	Near	10.97	High	51.06	3 052.00	147	NRP	11 362.00	8	Burkholderiales	Burkholderiaceae	Burkholderia
USA	*C. rohweri*	Bin 12	Bin 12_1	85.06	Substantial	22.88	Very high	39.13	3 421.00	202	NRP	23 818.00	25	Burkholderiales	Burkholderiaceae	Burkholderia
USA	*C. rohweri*	Bin 14	Bin 14_1	63.79	Moderate	17.82	Very high	91.89	2 045.00	121	NRP	16 730.00	11	Burkholderiales	Alcaligenaceae	Alcaligenes
USA	*C. rohweri*	Bin 14	Bin 14_2	63.79	Moderate	17.82	Very high	91.89	2 045.00	121	NRP	15 915.00	13	Burkholderiales	Alcaligenaceae	Alcaligenes
USA	*C. rohweri*	Bin 20	Bin 20	22.42	Partial	8.31	Medium	0.00	46 227.00	14 539.00	NRP	8 155.00	5	Burkholderiales	Alcaligenaceae	Alcaligenes
USA	*C. rohweri*	Bin 12	Bin 12_2	85.06	Substantial	22.88	Very high	39.13	3 421.00	202	NRP	16 717.00	7	Pseudomonadales	Ventosimonadaceae	Ventosimonas
USA	*C. rohweri*	Bin 10	Bin 10_2	98.28	Near	0.00	Low	0.00	2 295.00	77	Resorcinol	23 616.00	23	Burkholderiales	Alcaligenaceae	Bordetella
USA	*C. rohweri*	Bin 9	Bin 9_4	79.31	Substantial	53.45	Very high	20.45	5 174.00	424	Resorcinol	12 547.00	12	Xanthomonadales	Xanthomonadaceae	Xanthomonas
USA	*C. rohweri*	Bin 5	Bin 5_4	98.28	Near	10.97	High	51.06	3 052.00	147	Siderophore	10 804.00	9	Burkholderiales	Alcaligenaceae	Achromobacter
USA	*C. rohweri*	Bin 10	Bin 10_3	98.28	Near	0.00	Low	0.00	2 295.00	77	T1PK	15 520.00	9	Burkholderiales	Alcaligenaceae	Bordetella
USA	*C. rohweri*	Bin 15	Bin 15_1	69.83	Moderate	21.47	Very high	68.42	2 775.00	131	T1PK	32 313.00	27	Burkholderiales	Alcaligenaceae	Bordetella
USA	*C. rohweri*	Bin 15	Bin 15_4	69.83	Moderate	21.47	Very high	68.42	2 775.00	131	T1PK	16 292.00	8	Burkholderiales	Alcaligenaceae	Bordetella
USA	*C. rohweri*	Bin 9	Bin 9_1	79.31	Substantial	53.45	Very high	20.45	5 174.00	424	Terpene	16 223.00	17	Burkholderiales	Alcaligenaceae	Bordetella
USA	*C. rohweri*	Bin 10	Bin 10_1	98.28	Near	0.00	Low	0.00	2 295.00	77	Terpene	20 894.00	22	Burkholderiales	Alcaligenaceae	Bordetella
USA	*C. rohweri*	Bin 11	Bin 11	100.00	Near	0.00	Low	0.00	2 004.00	31	Terpene	15 849.00	13	Burkholderiales	Alcaligenaceae	Bordetella
USA	*C. rohweri*	Bin 16	Bin 16	88.09	Substantial	0.00	Low	0.00	2 073.00	90	Terpene	12 767.00	10	Burkholderiales	Alcaligenaceae	Bordetella
USA	*C. rohweri*	Bin 4	Bin 4_1	67.24	Moderate	12.07	High	4.35	18.614	3 378.00	Terpene	10 327.00	9	Rhizobiales	Bartonellaceae	Bartonella
USA	*C. rohweri*	Bin 5	Bin 5_1	98.28	Near	10.97	High	51.06	3 052.00	147	Terpene	12 715.00	12	Rhizobiales	Brucellaceae	Ochrobactrum
USA	*C. rohweri*	Bin 6	Bin 6	93.10	Near	0.00	Low	0.00	2 164.00	59	Terpene	18 554.00	18	Rhizobiales	Rhizobiaceae	Rhizobium

USA	*C. rohweri*	Bin 7	Bin 7	87.30	Substantial	2.30	Low	50.00	2 148.00	108	Terpene	19 007.00	18	Rhizobiales	Bartonellaceae	Bartonella
Peru	*C. similimus*	Bin 5	Bin 5_3	60.27	Moderate	21.00	Very high	12.50	803	4 703.00	Arylpolyene	5 313.00	5	Neisseriales	Neisseriaceae	Neisseria
Peru	*C. similimus*	Bin 6	Bin 6_6	75.39	Substantial	24.50	Very high	7.32	826	4 667.00	Arylpolyene	7 127.00	8	Burkholderiales	Alcaligenaceae	Achromobacter
Peru	*C. similimus*	Bin 5	Bin 5_1	60.27	Moderate	21.00	Very high	12.50	803	4 703.00	Arylpolyene	6 645.00	7	Burkholderiales	Alcaligenaceae	Achromobacter
Peru	*C. similimus*	Bin 6	Bin 6_4	75.39	Substantial	24.50	Very high	7.32	826	4 667.00	Arylpolyene	7 790.00	9	Xanthomonadales	Xanthomonadaceae	Xanthomonas
Peru	*C. similimus*	Bin 6	Bin 6_5	75.39	Substantial	24.50	Very high	7.32	826	4 667.00	Butyrolactone	6 364.00	8	Aeromonadales	Aeromonadaceae	Aeromonas
Peru	*C. similimus*	Bin 6	Bin 6_2	75.39	Substantial	24.50	Very high	7.32	826	4 667.00	Furan	5 440.00	9	Pseudomonadales	Pseudomonadaceae	Pseudomonas
Peru	*C. similimus*	Bin 5	Bin 5_2	60.27	Moderate	21.00	Very high	12.50	803	4 703.00	Lanthipeptide	8 781.00	8	Flavobacteriales	Flavobacteriaceae	Chryseobacterium
Peru	*C. spinosus*	Bin 2	Bin 2_2	87.93	Substantial	8.97	Medium	0.00	450	3 359.00	Arylpolyene	16 285.00	16	Burkholderiales	Alcaligenaceae	Bordetella
Peru	*C. spinosus*	Bin 2	Bin 2_4	87.93	Substantial	8.97	Medium	0.00	450	3 359.00	Arylpolyene	7 544.00	8	Burkholderiales	Alcaligenaceae	Achromobacter
Peru	*C. spinosus*	Bin 3	Bin 3	77.89	Substantial	18.97	Very high	75.00	267	2 519.00	Arylpolyene	25 079.00	26	Burkholderiales	Alcaligenaceae	Achromobacter
Peru	*C. spinosus*	Bin 4	Bin 4_2	41.38	Partial	5.17	Medium	33.33	173	2 059.00	Arylpolyene	6 935.00	5	Burkholderiales	Alcaligenaceae	Achromobacter
Peru	*C. spinosus*	Bin 5	Bin 5_1	98.28	Near	0.00	Low	0.00	81	2 658.00	Arylpolyene	41 203.00	44	Burkholderiales	Comamonadaceae	Acidovorax
Peru	*C. spinosus*	Bin 5	Bin 5_2	98.28	Near	0.00	Low	0.00	81	2 658.00	Arylpolyene	41 206.00	43	Burkholderiales	Comamonadaceae	Melaminivora
Peru	*C. spinosus*	Bin 8	Bin 8_1	93.18	Near	0.00	Low	0.00	825	3 903.00	Arylpolyene	5 023.00	8	Campylobacterales	Campylobacteraceae	Arcobacter
Peru	*C. spinosus*	Bin 7	Bin 7_1	100.00	Near	0.00	Low	0.00	514	4 141.00	Arylpolyene	36 717.00	32	Rhizobiales	Rhizobiaceae	Rhizobium
Peru	*C. spinosus*	Bin 8	Bin 8_2	93.18	Near	0.00	Low	0.00	825	3 903.00	Betalactone	14 713.00	13	Campylobacterales	Campylobacteraceae	Arcobacter
Peru	*C. spinosus*	Bin 6	Bin 6_1	54.20	Moderate	18.97	Very high	33.33	1 067.00	5 996.00	Butyrolactone	5 440.00	6	Burkholderiales	Comamonadaceae	Verminephrobacter
Peru	*C. spinosus*	Bin 2	Bin 2_1	87.93	Substantial	8.97	Medium	0.00	450	3 359.00	Ectoine	9 895.00	11	Burkholderiales	Alcaligenaceae	Pigmentiphaga
Peru	*C. spinosus*	Bin 6	Bin 6_4	54.20	Moderate	18.97	Very high	33.33	1 067.00	5 996.00	Lanthipeptide	6 371.00	4	Xanthomonadales	Xanthomonadaceae	Stenotrophomonas
Peru	*C. spinosus*	Bin 6	Bin 6_3	54.20	Moderate	18.97	Very high	33.33	1 067.00	5 996.00	NRP	12 639.00	12	Actinomycetales	Streptomycetaceae	Streptomyces
Peru	*C. spinosus*	Bin 6	Bin 6_5	54.20	Moderate	18.97	Very high	33.33	1 067.00	5 996.00	NRP	7 901.00	2	Pseudomonadales	Pseudomonadaceae	Pseudomonas
Peru	*C. spinosus*	Bin 6	Bin 6_7	54.20	Moderate	18.97	Very high	33.33	1 067.00	5 996.00	NRP	6 715.00	7	Pseudomonadales	Pseudomonadaceae	Pseudomonas
Peru	*C. spinosus*	Bin 6	Bin 6_2	54.20	Moderate	18.97	Very high	33.33	1 067.00	5 996.00	Phenazine	14 940.00	14	Pseudomonadales	Pseudomonadaceae	Pseudomonas
Peru	*C. spinosus*	Bin 7	Bin 7_2	100.00	Near	0.00	Low	0.00	514	4 141.00	Siderophore	7 469.00	4	Rhizobiales	Rhizobiaceae	Sinorhizobium
Peru	*C. spinosus*	Bin 7	Bin 7_3	100.00	Near	0.00	Low	0.00	514	4 141.00	Siderophore	8 038.00	6	Rhizobiales	Rhizobiaceae	Sinorhizobium
Peru	*C. spinosus*	Bin 2	Bin 2_3	87.93	Substantial	8.97	Medium	0.00	450	3 359.00	Terpene	21 709.00	21	Burkholderiales	Alcaligenaceae	Bordetella
Peru	*C. spinosus*	Bin 5	Bin 5_4	98.28	Near	0.00	Low	0.00	81	2 658.00	Terpene	5 320.00	5	Burkholderiales	Comamonadaceae	Acidovorax
Peru	*C. umbraculatus*	Bin 2	Bin 2_1	63.79	Moderate	30.38	Very high	3.12	664	3 383.00	Arylpolyene	26 451.00	31	Burkholderiales	Alcaligenaceae	Achromobacter
Peru	*C. umbraculatus*	Bin 5	Bin 5	98.28	Near	21.00	Very high	0.00	4 233.00	11 471.00	CDPS	9 839.00	15	Bdellovibrionales	Bdellovibrionaceae	Micavibrio

Peru	*C. umbraculatus*	Bin 3	Bin 3_2	93.03	Near	3.45	Low	33.33	332	4 114.00	Arylpolyene	34 176.00	36	Burkholderiales	Comamonadaceae	Delftia
Peru	*C. umbraculatus*	Bin 8	Bin 8_1	79.31	Substantial	14.55	High	0.00	669	3 307.00	Arylpolyene	32 683.00	27	Flavobacteriales	Crocinitomicaceae	Fluviicola
Peru	*C. umbraculatus*	Bin 8	Bin 8_2	79.31	Substantial	14.55	High	0.00	669	3 307.00	Arylpolyene	27 994.00	27	Flavobacteriales	Crocinitomicaceae	Fluviicola
Peru	*C. umbraculatus*	Bin 9	Bin 9_1	94.83	Near	2.35	Low	0.00	753	4 373.00	Arylpolyene	21 416.00	27	Flavobacteriales	Crocinitomicaceae	Fluviicola
Peru	*C. umbraculatus*	Bin 9	Bin 9_2	94.83	Near	2.35	Low	0.00	753	4 373.00	Arylpolyene	27 532.00	28	Flavobacteriales	Flavobacteriaceae	Elizabethkingia
Peru	*C. umbraculatus*	Bin 12	Bin 12	76.49	Substantial	50.00	Very high	15.38	374	2 580.00	Arylpolyene	33 424.00	32	Flavobacteriales	Flavobacteriaceae	Chryseobacterium
Peru	*C. umbraculatus*	Bin 4	Bin 4_2	100.00	Near	0.86	Low	0.00	147	2 493.00	Arylpolyene	26 803.00	30	Pseudomonadales	Pseudomonadaceae	Pseudomonas
Peru	*C. umbraculatus*	Bin 4	Bin 4_1	100.00	Near	0.86	Low	0.00	147	2 493.00	Arylpolyene-Ladderane	43 125.00	38	Pseudomonadales	Pseudomonadaceae	Pseudomonas
Peru	*C. umbraculatus*	Bin 3	Bin 3_4	93.03	Near	3.45	Low	33.33	332	4 114.00	NRP	7 797.00	6	Bacillales	Paenibacillaceae	Brevibacillus
Peru	*C. umbraculatus*	Bin 3	Bin 3_3	93.03	Near	3.45	Low	33.33	332	4 114.00	NRP	12 405.00	14	Pseudomonadales	Moraxellaceae	Acinetobacter
Peru	*C. umbraculatus*	Bin 2	Bin 2_2	63.79	Moderate	30.38	Very high	3.12	664	3 383.00	Phenazine	7 847.00	12	Xanthomonadales	Xanthomonadaceae	Lysobacter
Peru	*C. umbraculatus*	Bin 3	Bin 3_1	93.03	Near	3.45	Low	33.33	332	4 114.00	Terpene	10 431.00	11	Burkholderiales	Comamonadaceae	Delftia
USA	*C. varians* PL005	Bin 6	Bin 6_4	88.97	Substantial	48.28	Very high	52.50	219	3 237.00	Arylpolyene	24 835.00	28	Burkholderiales	Alcaligenaceae	Achromobacter
USA	*C. varians* PL005	Bin 4	Bin 4_1	98.28	Near	1.72	Low	100.00	253	2 957.00	Arylpolyene	31 597.00	34	Pseudomonadales	Ventosimonadaceae	Ventosimonas
USA	*C. varians* PL005	Bin 10	Bin 10_2	87.93	Substantial	34.48	Very high	6.90	583	3 581.00	Arylpolyene	17 255.00	22	Pseudomonadales	Pseudomonadaceae	Pseudomonas
USA	*C. varians* PL005	Bin 3	Bin 3_1	85.58	Substantial	57.76	Very high	45.24	459	3 650.00	Arylpolyene	8 577.00	8	Rhizobiales	Bartonellaceae	Bartonella
USA	*C. varians* PL005	Bin 10	Bin 10_1	87.93	Substantial	34.48	Very high	6.90	583	3 581.00	Arylpolyene	5 347.00	5	Xanthomonadales	Xanthomonadaceae	Stenotrophomonas
USA	*C. varians* PL005	Bin 1	Bin 1	98.28	Near	2.38	Low	0.00	1 020.00	4 203.00	Betalactone	20 310.00	17	Campylobacterales	Campylobacteraceae	Arcobacter
USA	*C. varians* PL005	Bin 4	Bin 4_3	98.28	Near	1.72	Low	100.00	253	2 957.00	Butyrolactone	6 403.00	9	Aeromonadales	Aeromonadaceae	Aeromonas
USA	*C. varians* PL005	Bin 11	Bin 11_1	46.08	Partial	31.03	Very high	64.00	480	3 651.00	Ladderane	7 506.00	10	Enterobacteriales	Enterobacteriaceae	Serratia
USA	*C. varians* PL005	Bin 11	Bin 11_2	46.08	Partial	31.03	Very high	64.00	480	3 651.00	Ladderane	18 686.00	19	Enterobacteriales	Enterobacteriaceae	Serratia
USA	*C. varians* PL005	Bin 9	Bin 9_1	79.31	Substantial	38.54	Very high	42.61	422	3 599.00	NRP	19 629.00	7	Bacillales	Paenibacillaceae	Brevibacillus
USA	*C. varians* PL005	Bin 4	Bin 4_4	98.28	Near	1.72	Low	100.00	253	2 957.00	NRP	8 444.00	7	Burkholderiales	Burkholderiaceae	Ralstonia
USA	*C. varians* PL005	Bin 5	Bin 5	89.66	Substantial	0.00	Low	0.00	67	1 793.00	NRP	28 568.00	25	Burkholderiales	Alcaligenaceae	Alcaligenes
USA	*C. varians* PL005	Bin 6	Bin 6_2	88.97	Substantial	48.28	Very high	52.50	219	3 237.00	NRP	30 680.00	27	Burkholderiales	Alcaligenaceae	Alcaligenes
USA	*C. varians* PL005	Bin 4	Bin 4_2	98.28	Near	1.72	Low	100.00	253	2 957.00	Siderophore	9 888.00	11	Burkholderiales	Alcaligenaceae	Achromobacter
USA	*C. varians* PL005	Bin 7	Bin 7	75.08	Substantial	41.69	Very high	58.82	224	2 656.00	T1PK	10 882.00	5	Burkholderiales	Alcaligenaceae	Achromobacter
USA	*C. varians* PL005	Bin 8	Bin 8	98.28	Near	26.80	Very high	3.85	120	2 542.00	T1PK	16 345.00	8	Burkholderiales	Alcaligenaceae	Achromobacter
USA	*C. varians* PL005	Bin 9	Bin 9_2	79.31	Substantial	38.54	Very high	42.61	422	3 599.00	Terpene	18 015.00	13	Burkholderiales	Comamonadaceae	Delftia
USA	*C. varians* PL005	Bin 9	Bin 9_3	79.31	Substantial	38.54	Very high	42.61	422	3 599.00	Terpene	14 521.00	11	Burkholderiales	Comamonadaceae	Delftia

USA	*C. varians PL010*	Bin 11	Bin_11_1	87.93	Substantial	0.00	Low	0.00	63	1845	Betalactone	20574	17	Campylobacterales	Campyrobacteraceae	Arcobacter
USA	*C. varians PL010*	Bin 9	Bin_9_1	94.83	Near	0.00	Low	0.00	84	2079	Resorcinol	20727	21	Xanthomonadales	Xanthomonadaceae	Stenotrophomonas
USA	*C. varians PL010*	Bin 7	Bin_7_1	98.28	Near	0.00	Low	0.00	136	1586	Terpene	15447	32	Burkholderiales	Comamonadaceae	Acidovorax
USA	*C. varians PL010*	Bin 7	Bin_7_2	98.28	Near	0.00	Low	0.00	136	1586	Terpene	11621	10	Burkholderiales	Comamonadaceae	Delftia

Supplementary Table 6. Genomic similarity measurement between metagenomic bins and cultured isolate genomes.

Host	Genome	Metagenomic bin	Order_identified	gANI	AF	gANI× AF	% of contigs mapped
C. rohweri	*Burkholderiales* sp. Cag20	Bin_5	Burkholderiales<br>Opitutales<br>Xanthomonadales	0.52	0.06	0.03	2.80
		Bin_8	Pseudomonadales	0.73	0.08	0.05	0.06
		Bin_9	Burkholderiales<br>Xanthomonadales	0.59	0.09	0.05	0.367
		Bin_10	Burkholderiales	0.55	0.15	0.08	0.03
		Bin_11	Burkholderiales	0.69	0.09	0.06	0.12
		Bin_12	Burkholderiales<br>Pseudomonadales	0.74	0.18	0.13	2.74
		Bin_13	Burkholderiales	0.70	0.21	0.14	0.92
		Bin_14	Burkholderiales	**0.97**	**0.85**	**0.82**	**41.86**
		Bin_15	Burkholderiales<br>Pseudomonadales	**0.98**	**0.87**	**0.85**	**53.65**
		Bin_16	Burkholderiales	0.74	0.08	0.05	0.06
		Bin_20	Burkholderiales	0	0	0	0.01
	Burkholderiales sp. Cag25	Bin_5	Burkholderiales<br>Opitutales<br>Xanthomonadales	0.51	0.09	0.04	0.03
		Bin_8	Pseudomonadales	0.64	0.12	0.07	0.03
		Bin_9	Burkholderiales<br>Xanthomonadales	0.51	0.14	0.07	0.03
		Bin_10	Burkholderiales	0.61	0.12	0.07	0.03
		Bin_11	Burkholderiales	0.52	0.17	0.08	0.06
		Bin_12	Burkholderiales<br>Pseudomonadales	0.83	0.12	0.09	13.38
		Bin_13	Burkholderiales	0.72	0.23	0.16	0.07
		Bin_14	Burkholderiales	**0.98**	**0.87**	**0.85**	**45.68**
		Bin_15	Burkholderiales<br>Pseudomonadales	**0.98**	**0.89**	**0.87**	**38.41**
		Bin_16	Burkholderiales	0.70	0.16	0.11	0.04
		Bin_20	Burkholderiales	0.00	0.00	0.00	0.00
	Cephaloticoccus primus Cag34	Bin_5	Burkholderiales<br>Opitutales<br>Xanthomonadales	0.88	0.02	0.01	2.12
		Bin_8	Pseudomonadales	0	0	0	0
		Bin_9	Burkholderiales<br>Xanthomonadales	0	0	0	0.01
		Bin_10	Burkholderiales	0	0	0	0
		Bin_11	Burkholderiales	0	0	0	0

		Bin_12	Burkholderiales Pseudomonadales	0	0	0	0
		Bin_13	Burkholderiales	0	0	0	0
		Bin_14	Burkholderiales	0.71	0.01	0	0
		Bin_15	Burkholderiales Pseudomonadales	0	0	0	0
		Bin_16	Burkholderiales	**0.96**	**0.82**	**0.78**	**35.59**
		Bin_20	Burkholderiales	0	0	0	0.06
	Ventosimonas sp. Cag26	Bin_5	Burkholderiales Opitutales Xanthomonadales	**0.96**	**0.80**	**0.76**	**11.30**
		Bin_8	Pseudomonadales	0.78	0.05	0.03	2.63
		Bin_9	Burkholderiales Xanthomonadales	0.71	0.04	0.02	0
		Bin_10	Burkholderiales	0.67	0.10	0.06	0
		Bin_11	Burkholderiales	0.72	0.08	0.05	0
		Bin_12	Burkholderiales Pseudomonadales	**0.99**	**0.89**	**0.88**	**65.37**
		Bin_13	Burkholderiales	0	0	0	0
		Bin_14	Burkholderiales	0.70	0.03	0.02	1.14
		Bin_15	Burkholderiales Pseudomonadales	0	0	0	0
		Bin_16	Burkholderiales	0.54	0.09	0.04	0
		Bin_20	Burkholderiales	0	0	0	0.03
	Ventosimonas sp. Cag27	Bin_5	Burkholderiales Opitutales Xanthomonadales	**0.96**	**0.80**	**0.76**	**11.31**
		Bin_8	Pseudomonadales	0.78	0.05	0.03	2.62
		Bin_9	Burkholderiales Xanthomonadales	0.71	0.04	0.02	0
		Bin_10	Burkholderiales	0.67	0.10	0.06	0
		Bin_11	Burkholderiales	0.72	0.08	0.05	0
		Bin_12	Burkholderiales Pseudomonadales	**0.99**	**0.89**	**0.88**	**65.49**
		Bin_13	Burkholderiales	0	0	0	0
		Bin_14	Burkholderiales	0.70	0.03	0.02	0.14
		Bin_15	Burkholderiales Pseudomonadales	0	0	0	0
		Bin_16	Burkholderiales	0.54	0.09	0.04	0
		Bin_20	Burkholderiales	0	0	0	0
	Ventosimonas sp. Cag320	Bin_5	Burkholderiales Opitutales Xanthomonadales	0.87	0	0	0
		Bin_8	Pseudomonadales	0.80	0.07	0.05	3.09
		Bin_9	Burkholderiales Xanthomonadales	0.64	0.05	0.03	1.03
		Bin_10	Burkholderiales	0.69	0.07	0.04	0
		Bin_11	Burkholderiales	0	0	0	0

		Bin_12	Burkholderiales<br>Pseudomonadales	**0.99**	**0.84**	**0.83**	**56.79**
		Bin_13	Burkholderiales	0	0	0	1.10
		Bin_14	Burkholderiales	0	0	0	0.01
		Bin_15	Burkholderiales<br>Pseudomonadales	0.61	0.04	0.02	0
		Bin_16	Burkholderiales	0.66	0.15	0.09	0
		Bin_20	Burkholderiales	0	0	0	1.19
	Xanthomonadales sp. Cag60	Bin_5	Burkholderiales<br>Opitutales<br>Xanthomonadales	0.74	0.03	0.02	0.96
		Bin_8	Pseudomonadales	0.69	0.08	0.05	0.11
		Bin_9	Burkholderiales<br>Xanthomonadales	**0.97**	**0.82**	**0.79**	**33.36**
		Bin_10	Burkholderiales	0.68	0.08	0.05	0.11
		Bin_11	Burkholderiales	0.71	0.04	0.02	0.01
		Bin_12	Burkholderiales<br>Pseudomonadales	0.76	0.05	0.03	0.89
		Bin_13	Burkholderiales	0.64	0.07	0.04	0.14
		Bin_14	Burkholderiales	0	0	0	0.07
		Bin_15	Burkholderiales<br>Pseudomonadales	0.65	0.05	0.03	0.02
		Bin_16	Burkholderiales	0.72	0.05	0.03	0
		Bin_20	Burkholderiales	0	0	0	0.01
C.varians PL005W	*Burkholderiales* sp. Cv33a	Bin_3	Rhizobiales	0.66	0.03	0.01	0.06
		Bin_4	Burkholderiales<br>Pseudomonadales	0.69	0.08	0.05	0.95
		Bin_5	Burkholderiales	0.66	0.15	0.09	0.10
		Bin_6	Burkholderiales	0.66	0.21	0.13	0.16
		Bin_7	Burkholderiales	0.68	0.12	0.08	0.04
		Bin_8	Burkholderiales	**0.98**	**0.93**	**0.91**	**83.26**
		Bin_9	Burkholderiales	0.70	0.20	0.14	4.153
		Bin_10	Pseudomonadales<br>Xanthomonadales	0.58	0.05	0.02	0.06
	Burkholderiales sp. Cv36	Bin_3	Rhizobiales	0	0	0	0
		Bin_4	Burkholderiales<br>Pseudomonadales	0.72	0.07	0.05	0.58
		Bin_5	Burkholderiales	0.61	0.14	0.08	0.25
		Bin_6	Burkholderiales	0.72	0.13	0.09	0.33
		Bin_7	Burkholderiales	0.64	0.12	0.07	0.60
		Bin_8	Burkholderiales	0.71	0.24	0.17	0.45
		Bin_9	Burkholderiales	0.78	0.10	0.07	1.07
		Bin_10	Pseudomonadales<br>Xanthomonadales	0.66	0.04	0.02	0.08
	Burkholderiales sp. Cv44	Bin_3	Rhizobiales	0	0	0	0.04
		Bin_4	Burkholderiales<br>Pseudomonadales	0.73	0.08	0.05	1.83
		Bin_5	Burkholderiales	0.74	0.15	0.11	1.13
		Bin_6	Burkholderiales	0.91	0.18	0.16	22.25

		Bin_7	Burkholderiales	**0.97**	**0.83**	**0.80**	**36.86**
		Bin_8	Burkholderiales	0.67	0.12	0.08	1.30
		Bin_9	Burkholderiales	0.86	0.14	0.12	14.08
		Bin_10	Pseudomonadales Xanthomonadales	0.54	0.05	0.02	1.04
	Burkholderiales sp. Cv52	Bin_3	Rhizobiales	0.64	0.04	0.02	0
		Bin_4	Burkholderiales Pseudomonadales	0.80	0.08	0.06	0.48
		Bin_5	Burkholderiales	0.61	0.14	0.08	0.21
		Bin_6	Burkholderiales	0.62	0.18	0.11	0.43
		Bin_7	Burkholderiales	0.74	0.16	0.11	0.30
		Bin_8	Burkholderiales	0.75	0.13	0.09	0.26
		Bin_9	Burkholderiales	0.77	0.13	0.10	1.13
		Bin_10	Pseudomonadales Xanthomonadales	0.58	0.05	0.02	0.05
	Cephaloticoccus capnophilus Cv41	Bin_3	Rhizobiales	0	0	0	0
		Bin_4	Burkholderiales Pseudomonadales	0.72	0.02	0.01	1.52
		Bin_5	Burkholderiales	0.57	0.12	0.06	0.00
		Bin_6	Burkholderiales	0.69	0.18	0.12	2.02
		Bin_7	Burkholderiales	0.56	0.09	0.05	2.02
		Bin_8	Burkholderiales	0	0	0	0
		Bin_9	Burkholderiales	0.73	0.04	0.02	2.26
		Bin_10	Pseudomonadales Xanthomonadales	**0.99**	**0.84**	**0.83**	**60.71**
	Rhizobiales sp. JR021-5	Bin_3	Rhizobiales	0	0	0	0
		Bin_4	Burkholderiales Pseudomonadales	0	0	0	0
		Bin_5	Burkholderiales	0	0	0	0
		Bin_6	Burkholderiales	0	0	0	0
		Bin_7	Burkholderiales	0	0	0	0
		Bin_8	Burkholderiales	0	0	0	0
		Bin_9	Burkholderiales	0	0	0	0
		Bin_10	Pseudomonadales Xanthomonadales	0	0	0	0
	Ventosimonas gracilis Cv58	Bin_3	Rhizobiales	0	0	0	0
		Bin_4	Burkholderiales Pseudomonadales	**0.96**	**0.84**	**0.80**	**49.31**
		Bin_5	Burkholderiales	0	0	0	0.05
		Bin_6	Burkholderiales	0.70	0.06	0.04	0.13
		Bin_7	Burkholderiales	0.68	0.06	0.04	0.28
		Bin_8	Burkholderiales	0.55	0.19	0.10	0.05
		Bin_9	Burkholderiales	0.76	0.08	0.06	0.84
		Bin_10	Pseudomonadales Xanthomonadales	0.61	0.05	0.03	0.11

C. varians		Bin_1	Burkholderiales	**1.00**	**0.85**	**0.85**	**98.48**

PL010W	Burkholderiales sp. Cv33a	Bin_2	Burkholderiales	0.70	0.19	0.13	0.04
		Bin_3	Rhizobiales	0	0	0	0.04
		Bin_4	Rhizobiales	0	0	0	0
		Bin_5	Opitutales	0.70	0.11	0.08	0.07
		Bin_7	Burkholderiales	0.65	0.10	0.07	0.04
		Bin_8	Rhizobiales	0.68	0.02	0.01	0.04
		Bin_9	Burkholderiales Pseudomonadales Xanthomonadales	0.67	0.09	0.06	0.12
	Burkholderiales sp. Cv36	Bin_1	Burkholderiales	0.70	0.15	0.11	0.05
		Bin_2	Burkholderiales	0.78	0.19	0.15	0.22
		Bin_3	Rhizobiales	0	0	0	0.04
		Bin_4	Rhizobiales	0	0	0	0
		Bin_5	Opitutales	0.52	0.08	0.04	0.02
		Bin_7	Burkholderiales	0.71	0.08	0.06	0
		Bin_8	Rhizobiales	0	0	0	0.02
		Bin_9	Burkholderiales Pseudomonadales Xanthomonadales	0.61	0.04	0.02	0.02
	Burkholderiales sp. Cv44	Bin_1	Burkholderiales	0.75	0.15	0.11	0.11
		Bin_2	Burkholderiales	0.74	0.16	0.12	0
		Bin_3	Rhizobiales	0	0	0	0.04
		Bin_4	Rhizobiales	0	0	0	0
		Bin_5	Opitutales	0.57	0.11	0.06	0.02
		Bin_7	Burkholderiales	0.69	0.08	0.06	0.08
		Bin_8	Rhizobiales	0.70	0.02	0.01	0.04
		Bin_9	Burkholderiales Pseudomonadales Xanthomonadales	0.58	0.05	0.03	0
	Burkholderiales sp. Cv52	Bin_1	Burkholderiales	0.76	0.12	0.09	0.11
		Bin_2	Burkholderiales	1.00	0.50	0.50	96.48
		Bin_3	Rhizobiales	0	0	0	0
		Bin_4	Rhizobiales	0	0	0	0
		Bin_5	Opitutales	0.65	0.16	0.10	0.17
		Bin_7	Burkholderiales	0.69	0.11	0.08	0.06
		Bin_8	Rhizobiales	0.66	0.02	0.01	0.02
		Bin_9	Burkholderiales Pseudomonadales Xanthomonadales	0.70	0.03	0.02	0.05
	Cephaloticoccus capnophilus Cv41	Bin_1	Burkholderiales	0	0	0	0.03
		Bin_2	Burkholderiales	0	0	0	0
		Bin_3	Rhizobiales	0	0	0	0
		Bin_4	Rhizobiales	0	0	0	0
		Bin_5	Opitutales	0.96	0.63	0.60	80.56
		Bin_7	Burkholderiales	0	0	0	0.06

		Bin_8	Rhizobiales	0	0	0	0.02
		Bin_9	Burkholderiales<br>Pseudomonadales<br>Xanthomonadales	0	0	0	0
	Rhizobiales sp. JR021-5	Bin_1	Burkholderiales	0.72	0.01	0.01	0
		Bin_2	Burkholderiales	0.70	0.01	0.01	0
		Bin_3	Rhizobiales	0	0	0	0
		Bin_4	Rhizobiales	0	0	0	0
		Bin_5	Opitutales	0	0	0	0
		Bin_7	Burkholderiales	0	0	0	0
		Bin_8	Rhizobiales	0	0	0	0
		Bin_9	Burkholderiales<br>Pseudomonadales<br>Xanthomonadales	0	0	0	0
	Ventosimonas gracilis Cv58	Bin_1	Burkholderiales	0.68	0.07	0.05	0.05
		Bin_2	Burkholderiales	0.68	0.09	0.06	0.04
		Bin_3	Rhizobiales	0	0	0	0.04
		Bin_4	Rhizobiales	0	0	0	0
		Bin_5	Opitutales	0.42	0.10	0.04	0.04
		Bin_7	Burkholderiales	0.69	0.08	0.06	0.10
		Bin_8	Rhizobiales	0.69	0.03	0.02	0.02
		Bin_9	Burkholderiales<br>Pseudomonadales<br>Xanthomonadales	0	0	0	0.24

Supplementary Table 7. Genomic similarity measurement between BGCs from cultured isolate bacterial genomes and metagenomic bins.

Host	Genome	BCG ID in genome	BGC type	Metagenomic bin	BCG ID in bin	Order identified	gANI	AF	gANI× AF	% of contigs mapped
C_rohweri	*Cephaloticoccus primus* Cag34	primus	Arylpolyene	Bin_4	Bin_4_2	Rhizobiales	0	0	0	0
				Bin_5	Bin_5_2	Rhizobiales	0	0	0	0
				Bin_5	Bin_5_5	Pseudomonadales	**0.71**	**0.45**	**0.31**	**36.53**
				Bin_8	Bin_8	Pseudomonadales	0	0	0	0
	Ventosimonas sp. Cag26 BGC1	Ventosimonas_26_1	Arylpolyene	Bin_4	Bin_4_2	Rhizobiales	0	0	0	0
				Bin_5	Bin_5_2	Rhizobiales	0	0	0	0
				Bin_5	Bin_5_5	Pseudomonadales	**1.00**	**0.98**	**0.98**	**100**
				Bin_8	Bin_8	Pseudomonadales	0	0	0	0
	Ventosimonas sp. Cag27 BGC2	Ventosimonas_27_2	Arylpolyene	Bin_4	Bin_4_2	Rhizobiales	0	0	0	0
				Bin_5	Bin_5_2	Rhizobiales	0	0	0	0
				Bin_5	Bin_5_5	Pseudomonadales	**1.00**	**0.98**	**0.98**	**100**
				Bin_8	Bin_8	Pseudomonadales	0	0	0	0
	Xanthomonadales sp. Cag60 BGC5	Xanthomonadales_60_5	Arylpolyene	Bin_4	Bin_4_2	Rhizobiales	0	0	0	0
				Bin_5	Bin_5_2	Rhizobiales	0	0	0	0
				Bin_5	Bin_5_5	Pseudomonadales	0	0	0	0
				Bin_8	Bin_8	Pseudomonadales	0	0	0	0
	Burkholderiales sp. Cag20 BGC2	Burkholderiales_20_2	NRP	Bin_5	Bin_5_3	Burkholderiales	0	0	0	0
				Bin_12	Bin_12_1	Burkholderiales	0	0	0	0
				Bin_12	Bin_12_2	Neisseriales	0	0	0	0
				Bin_14	Bin_14_1	Burkholderiales	**1.00**	**0.98**	**0.98**	**100**
				Bin_14	Bin_14_2	Burkholderiales	0	0	0	0
				Bin_20	Bin_20	Burkholderiales	0	0	0	0
	Burkholderiales sp. Cag20 BGC4	Burkholderiales_20_4	NRP	Bin_5	Bin_5_3	Burkholderiales	0	0	0	0
				Bin_12	Bin_12_1	Burkholderiales	0	0	0	0
				Bin_12	Bin_12_2	Neisseriales	0	0	0	0
				Bin_14	Bin_14_1	Burkholderiales	0	0	0	0
				Bin_14	Bin_14_2	Burkholderiales	0	0	0	0

				Bin_20	Bin_20	Burkholderiales	0	0	0	0
	Burkholderiales sp. Cag20 BGC5	Burkholderiales_20_5	NRP	Bin_5	Bin_5_3	Burkholderiales	0	0	0	0
				Bin_12	Bin_12_1	Burkholderiales	0	0	0	0
				Bin_12	Bin_12_2	Neisseriales	0	0	0	0
				Bin_14	Bin_14_1	Burkholderiales	0	0	0	0
				Bin_14	Bin_14_2	Burkholderiales	0	0	0	0
				Bin_20	Bin_20	Burkholderiales	0	0	0	0
	Burkholderiales sp. Cag25 BGC3	Burkholderiales_25_3	NRP	Bin_5	Bin_5_3	Burkholderiales	0	0	0	0
				Bin_12	Bin_12_1	Burkholderiales	0	0	0	0
				Bin_12	Bin_12_2	Neisseriales	0	0	0	0
				Bin_14	Bin_14_1	Burkholderiales	0	0	0	0
				Bin_14	Bin_14_2	Burkholderiales	**0.99**	**0.98**	**0.97**	**100**
				Bin_20	Bin_20	Burkholderiales	0	0	0	0
	Burkholderiales sp. Cag25 BGC4	Burkholderiales_25_4	NRP	Bin_5	Bin_5_3	Burkholderiales	0	0	0	0
				Bin_12	Bin_12_1	Burkholderiales	0	0	0	0
				Bin_12	Bin_12_2	Neisseriales	0	0	0	0
				Bin_14	Bin_14_1	Burkholderiales	0	0	0	0
				Bin_14	Bin_14_2	Burkholderiales	0	0	0	0
				Bin_20	Bin_20	Burkholderiales	0	0	0	0
	Ventosimonas sp. Cag26 BGC2	Ventosimonas_26_2	NRP	Bin_5	Bin_5_3	Burkholderiales	0	0	0	0
				Bin_12	Bin_12_1	Burkholderiales	0	0	0	0
				Bin_12	Bin_12_2	Pseudomonadales	**0.96**	**0.86**	**0.82**	**92.65**
				Bin_14	Bin_14_1	Burkholderiales	0	0	0	0
				Bin_14	Bin_14_2	Burkholderiales	0	0	0	0
				Bin_20	Bin_20	Burkholderiales	0	0	0	0
	Ventosimonas sp. Cag27 BGC1	Ventosimonas_27_1	NRP	Bin_5	Bin_5_3	Burkholderiales	0	0	0	0
				Bin_12	Bin_12_1	Burkholderiales	0	0	0	0
				Bin_12	Bin_12_2	Pseudomonadales	**0.96**	**0.82**	**0.78**	**92.53**
				Bin_14	Bin_14_1	Burkholderiales	0	0	0	0
				Bin_14	Bin_14_2	Burkholderiales	0	0	0	0
				Bin_20	Bin_20	Burkholderiales	0	0	0	0
		Ventosimonas_320_1	NRP	Bin_5	Bin_5_3	Burkholderiales	0	0	0	0

	Ventosimonas sp. Cag320 BGC1			Bin_12	Bin_12_1	Burkholderiales	0	0	0	0
				Bin_12	Bin_12_2	Neisseriales	0	0	0	0
				Bin_14	Bin_14_1	Burkholderiales	0	0	0	0
				Bin_14	Bin_14_2	Burkholderiales	0	0	0	0
				Bin_20	Bin_20	Burkholderiales	0	0	0	0
	Ventosimonas sp. Cag320 BGC2	Ventosimonas_320_2	NRP	Bin_5	Bin_5_3	Burkholderiales	0	0	0	0
				Bin_12	Bin_12_1	Burkholderiales	0	0	0	0
				Bin_12	Bin_12_2	Neisseriales	0	0	0	0
				Bin_14	Bin_14_1	Burkholderiales	0	0	0	0
				Bin_14	Bin_14_2	Burkholderiales	0	0	0	0
				Bin_20	Bin_20	Burkholderiales	0	0	0	0
	Xanthomonadales sp. Cag60 BGC1	Xanthomonadales_60_1	NRP	Bin_5	Bin_5_3	Burkholderiales	0	0	0	0
				Bin_12	Bin_12_1	Burkholderiales	0	0	0	0
				Bin_12	Bin_12_2	Neisseriales	0	0	0	0
				Bin_14	Bin_14_1	Burkholderiales	0	0	0	0
				Bin_14	Bin_14_2	Burkholderiales	0	0	0	0
				Bin_20	Bin_20	Burkholderiales	0	0	0	0
	Xanthomonadales sp. Cag60 BGC3	Xanthomonadales_60_3	NRP	Bin_5	Bin_5_3	Burkholderiales	0	0	0	0
				Bin_12	Bin_12_1	Burkholderiales	0	0	0	0
				Bin_12	Bin_12_2	Neisseriales	0	0	0	0
				Bin_14	Bin_14_1	Burkholderiales	0	0	0	0
				Bin_14	Bin_14_2	Burkholderiales	0	0	0	0
				Bin_20	Bin_20	Burkholderiales	0	0	0	0
	Xanthomonadales sp. Cag60 BGC4	Xanthomonadales_60_4	Siderophore	Bin_5	Bin_5_4	Burkholderiales	0	0	0	0
	Xanthomonadales sp. Cag60 BGC2	Xanthomonadales_60_2	Ladderane	Bin_9	Bin_9_2	Xanthomonadales	**0.96**	**0.52**	**0.49**	**100**
				Bin_9	Bin_9_3	Enterobacteriales	0	0	0	0
	Burkholderiales sp. Cag20 BGC1	Burkholderiales_20_1	T1PK	Bin_10	Bin_10_3	Burkholderiales	0	0	0	0
				Bin_15	Bin_15_1	Burkholderiales	**1.00**	**0.98**	**0.98**	**100**
				Bin_15	Bin_15_4	Burkholderiales	**0.80**	**0.39**	**0.31**	**54.37**
	Burkholderiales sp. Cag25 BGC2	Burkholderiales_25_2 Burkholderiales_20_3	T1PK	Bin_10	Bin_10_3	Burkholderiales	0	0	0	0
				Bin_15	Bin_15_1	Burkholderiales	0	0	0	0
				Bin_15	Bin_15_4	Burkholderiales	**0.99**	**0.98**	**0.97**	**90.90**

	Burkholderiales sp. Cag20 BGC3		Betalactone	Bin_12	Bin_12_3	Pseudomonadales	0	0	0	0
				Bin_13	Bin_13	Burkholderiales	0	0	0	0
				Bin_15	Bin_15_2	Pseudomonadales	0	0	0	0
				Bin_15	Bin_15_3	Burkholderiales	**1.00**	**0.98**	**0.98**	**100**
	Burkholderiales sp. Cag25 BGC1	Burkholderiales_25_1	Betalactone	Bin_12	Bin_12_3	Pseudomonadales	0	0	0	0
				Bin_13	Bin_13	Burkholderiales	0	0	0	0
				Bin_15	Bin_15_2	Pseudomonadales	0	0	0	0
				Bin_15	Bin_15_3	Burkholderiales	**0.99**	**0.98**	**0.97**	**97.22**
C_varian _005	Burkholderiales sp. Cv44 BGC3	Burkholderiales_44_3	Betalactone	Bin_1	Bin_1	Campylobacterales	0	0	0	0
	Burkholderiales sp. Cv36 BGC2	Burkholderiales_36_2	Arylpolyene	Bin_3	Bin_3_1	Rhizobiales	0	0	0	0
				Bin_4	Bin_4_1	Pseudomonadales	0	0	0	0
				Bin_6	Bin_6_4	Burkholderiales	0	0	0	0
				Bin_10	Bin_10_1	Xanthomonadales	0	0	0	0
				Bin_10	Bin_10_2	Pseudomonadales	0	0	0	0
	Ventosimonas gracilis Cv58	Ventosimonas_58	Arylpolyene	Bin_3	Bin_3_1	Rhizobiales	0	0	0	0
				Bin_4	Bin_4_1	Pseudomonadales	**0.97**	**0.80**	**0.77**	**93.12**
				Bin_6	Bin_6_4	Burkholderiales	0	0	0	0
				Bin_10	Bin_10_1	Xanthomonadales	0	0	0	0
				Bin_10	Bin_10_2	Pseudomonadales	0	0	0	0
	Burkholderiales sp. Cv44 BGC2	Burkholderiales_44_2	NRP	Bin_4	Bin_4_4	Burkholderiales	0	0	0	0
				Bin_5	Bin_5	Burkholderiales	0	0	0	0
				Bin_6	Bin_6_2	Burkholderiales	0	0	0	0
				Bin_9	Bin_9_1	Burkholderiales	0	0	0	0
	Burkholderiales sp. Cv44 BGC1	Burkholderiales_44_1	T1PK	Bin_7	Bin_7	Burkholderiales	**1.00**	**0.98**	**0.98**	**100**
				Bin_8	Bin_8	Burkholderiales	0	0	0	0
	Burkholderiales sp. Cv52 BGC3	Burkholderiales_52_3	T1PK	Bin_7	Bin_7	Burkholderiales	**0.80**	**0.49**	**0.39**	**47.82**
				Bin_8	Bin_8	Burkholderiales	0	0	0	0
	Burkholderiales sp. Cv36 BGC1	Burkholderiales_36_1	Terpene	Bin_9	Bin_9_2	Burkholderiales	0	0	0	0
	Burkholderiales sp. Cv52 BGC1	Burkholderiales_52_1	Terpene	Bin_9	Bin_9_2	Burkholderiales	0	0	0	0

C_varians _010	Burkholderiales_36_1	Burkholderiales_36_1	Terpene	Bin_7	Bin_7_1	Burkholderiales	0	0	0	0
				Bin_7	Bin_7_2	Burkholderiales	0	0	0	0
	Burkholderiales_52_1	Burkholderiales_52_1	Terpene	Bin_7	Bin_7_1	Burkholderiales	0	0	0	0
				Bin_7	Bin_7_2	Burkholderiales	0	0	0	0

In this table, we only consider the BGC types which were found in the genomes. If a BGC type was identified in a metagenomic bin of a specific *Cephalotes* species, but not in a genome of this same *Cephalotes* species, the BGC will not be taken into account and will not appear in the table.

Supplementary Table 8. Reference BGC and query protein selected in the CORASON phylogenetic analysis.

Type of BGC	Reference BGC	Reference BGC length (bp)	Reference BGC length (gene number)	Query protein domain	Query protein coordinates in the reference BGC
Arylpolyene	*C. spinosus* Bin_5_1	41,203	44	APE_KS2	38,905-40,107
NRP	*C. rohweri* Xanthomonadales Cag60_3	53,609	45	AMP-binding (adenylation domain)	64,471-65,991
T1PK	*C. rohweri* Burkholderiales Cag25_2	47,911	35	PKS_KS	197,260-205,170
Siderophore	*C. pellans* Bin_2_7	9,526	11	IucA_IucC	2,926-4,788
